# Genetic Diversity and Gene Family Expansions in Members of the Genus *Entamoeba*

**DOI:** 10.1093/gbe/evz009

**Published:** 2019-01-21

**Authors:** Ian W Wilson, Gareth D Weedall, Hernan Lorenzi, Timothy Howcroft, Chung-Chau Hon, Marc Deloger, Nancy Guillén, Steve Paterson, C Graham Clark, Neil Hall

**Affiliations:** 1 Institute of Integrative Biology, University of Liverpool, United Kingdom; 2 School of Natural Sciences and Psychology, Liverpool John Moores University, United Kingdom; 3 J. Craig Venter Institute, Rockville, Maryland; 4 Unité Biologie Cellulaire du Parasitisme, Institut Pasteur, Paris, France; 5 London School of Hygiene & Tropical Medicine, Faculty of Infectious and Tropical Diseases, London, United Kingdom; 6 Earlham Institute, Norwich Research Park, Norwich, United Kingdom; 7 School of Biological Sciences, University of East Anglia, Norwich Research Park, Norwich, NR4 7TJ United Kingdom

**Keywords:** *Entamoeba*, gene family, genome diversity, species complex

## Abstract

Amoebiasis is the third-most common cause of mortality worldwide from a parasitic disease. Although the primary etiological agent of amoebiasis is the obligate human parasite *Entamoeba histolytica*, other members of the genus *Entamoeba* can infect humans and may be pathogenic. Here, we present the first annotated reference genome for *Entamoeba moshkovskii*, a species that has been associated with human infections, and compare the genomes of *E. moshkovskii*, *E. histolytica*, the human commensal *Entamoeba dispar*, and the nonhuman pathogen *Entamoeba invadens*. Gene clustering and phylogenetic analyses show differences in expansion and contraction of families of proteins associated with host or bacterial interactions. They intimate the importance to parasitic *Entamoeba* species of surface-bound proteins involved in adhesion to extracellular membranes, such as the Gal/GalNAc lectin and members of the BspA and Ariel1 families. Furthermore, *E. dispar* is the only one of the four species to lack a functional copy of the key virulence factor cysteine protease *CP-A5*, whereas the gene’s presence in *E. moshkovskii* is consistent with the species’ potentially pathogenic nature. *Entamoeba moshkovskii* was found to be more diverse than *E. histolytica* across all sequence classes. The former is ∼200 times more diverse than latter, with the four *E. moshkovskii* strains tested having a most recent common ancestor nearly 500 times more ancient than the tested *E. histolytica* strains. A four-haplotype test indicates that these *E. moshkovskii* strains are not the same species and should be regarded as a species complex.

## Introduction

Amoebiasis affects up to 50 million people annually, resulting in up to 100,000 deaths (Walsh 1986). The etiological agent of amoebiasis in humans is the obligate human parasite *Entamoeba histolytica*, which is transmitted between hosts by a fecal–oral route. The outcome of infection ranges from asymptomatic carriage (in the majority of cases) to dysentery, characterized by bloody stools and, in some cases where parasites escape the gut, abscesses in the liver and other organs that are fatal if untreated. Amoebiasis is particularly prevalent in areas of poor sanitation, and people living in these conditions are the most commonly affected. Outside of these settings, risk groups are travelers returning from endemic regions, people who engage in risky sexual practices (Stark et al. 2007, 2008) and institutionalized populations (Rivera et al. 2006; Nishise et al. 2010).

The low proportion of infections that result in invasive amoebiasis remains unexplained. Our understanding of the epidemiology of the disease was complicated, in part, by the existence of a second, noninvasive, member of the genus *Entamoeba*—*Entamoeba dispar* (Diamond and Clark 1993). Morphologically identical to *E. histolytica* and closely related, *E. dispar* is infective to humans but is thought to be avirulent (Diamond and Clark 1993; Bansal et al. 2009) despite liver-derived clinical isolates of *E. dispar* bringing its avirulence into question (Ximénez et al. 2010). Invasive disease is deleterious to the parasite as trophozoites passing into the blood or tissues will not go on to form cysts and infect new hosts. Therefore, “virulence” should not be selected for and may be considered as a negative interaction for the host and parasite.

The differences in virulence capabilities seen between *E. dispar* and *E. histolytica* have been exploited by various groups attempting to determine which proteins may enable virulence capabilities in *E. histolytica* but not in *E. dispar* (Leitsch et al. 2006; Davis et al. 2009). Two key families, which we investigate here in relation to host–parasite interactions in a greater number of *Entamoeba* species, are the cysteine proteases and the Gal/GalNAc lectin proteins.

To invade the intestinal epithelium, trophozoites must first degrade and cross the mucosal layer that covers and protects it. The cysteine proteases are a group of at least 50 endopeptidases, 36 of which form three major clades—“A,” “B,” and “C” (Clark et al. 2007; Casados-Vázquez et al. 2011). Although, collectively, the cysteine proteases are regarded as virulence factors, evidence suggests that ∼90% of *E. histolytica’*s cysteine protease-derived proteolytic activity is provided by just three proteins—*EhCP-A1*, *EhCP-A2*, and *EhCP-A5* (Stanley et al. 1995; Bruchhaus et al. 1996; Ankri et al. 1999; Meléndez-López et al. 2007). *EhCP-A5* is of particular interest as no functional ortholog exists in the nonpathogenic *E. dispar* (Jacobs et al. 1998) and expression of the protein is thought to be necessary for *E. histolytica* to invade the human intestinal mucosa (Thibeaux et al. 2014). In concert with amoebic glycosidases, an undefined number of cysteine proteases degrade the *MUC2* polymers that constitute much of the mucosal layer (Moncada et al. 2003, 2005). Trophozoites employ surface-bound proteins to bind to host mucins as a natural part of a commensal lifecycle and, once they have degraded the mucosal layer, epithelial cells. One such protein is the Gal/GalNAc lectin, a heterodimer comprising a 170-kDa heavy subunit and a 35-kDa light subunit, associated with a 150-kDa intermediate subunit (Petri et al. 2002). The lectin binds to galactose and *N*-acetyl-d-galactosamine on host cell membranes. Without it, *E. histolytica’*s ability to adhere to host cells is significantly diminished, as is its cytotoxic impact upon the host cells, leading to the understanding that the cytokine cascade induced by *E. histolytica* that ultimately leads to the degradation of host cells is contact-dependent (Li et al. 1988, 1989; Ravdin et al. 1980, 1989; Stanley 2003). However, despite the wealth of knowledge that exists regarding gene families potentially responsible for causing invasive amoebiasis such as the cysteine proteases and Gal/GalNAc lectins, much uncertainty remains regarding which of these families play essential roles and what key differences exist between those species and strains capable of causing pathology and those that cannot.

A more distantly related species, *Entamoeba moshkovskii*, was originally thought to be free-living and therefore nonpathogenic (Tshalaia 1941; Neal 1953; Clark and Diamond 1997). However, as with *E. dispar*, human-derived clinical isolates (Clark and Diamond 1991) and cases of diarrhea directly associated with *E. moshkovskii* infection (Fotedar et al. 2008; Shimokawa et al. 2012) have challenged this assumption. As such, the ability of *E. moshkovskii* to cause invasive amoebiasis is of increasing interest, with multiple studies presenting further evidence that *E. moshkovskii* is human-infective and potentially pathogenic (Hamzah et al. 2006; Khairnar and Parija 2007; Ayed et al. 2008; ElBakri et al. 2013; Lau et al. 2013).

Despite its evolutionary distance from *E. histolytica*, *E. dispar*, and *E. moshkovskii* (Stensvold et al. 2011), the reptile-infective *Entamoeba invadens* is also known to be pathogenic and can cause fatal disease in a wide range of reptiles (Meerovitch 1958; Kojimoto et al. 2001; Chia et al. 2009). This species is also of interest for research into lifecycle development because it is the only member of the genus for which encystation can be successfully induced in vitro in axenic culture, using various methods (Vázquezdelara-Cisneros and Arroyo-Begovich 1984; Avron et al. 1986; García-Zapién et al. 1995). Through genome sequencing, it was found that *E. invadens* has an average sequence identity with *E. histolytica* of 60% (Wang et al. 2003; Ehrenkaufer 2013).

Several reports, focusing on single nucleotide polymorphisms (SNPs), have found evidence to support the theory of limited genetic diversity among *E. histolytica* strains (Beck et al. 2002; Bhattacharya et al. 2005; Weedall et al. 2012). Initially, this was thought to indicate a clonal species; however, evidence of meiotic recombination has been discovered, suggesting that *E. histolytica* actually reproduces sexually (Weedall et al. 2012). There is a relative paucity of studies into diversity in other members of the genus *Entamoeba*. In the case of *E. moshkovskii*, this is because, until now, there was no reference genome with which to compare different strains. In spite of this, there is support for the theory that *E. moshkovskii* is, in fact, highly variable and may be a species complex, rather than an individual species (Clark and Diamond 1997; Jacob et al. 2016). If we are able to more accurately identify which isolates are capable of infecting humans or causing disease, it may afford us a greater understanding of the genetic and molecular mechanisms behind these traits.

Here, we present the first annotated genome for *E. moshkovskii*. We have compared this with the sequenced genomes of other members of the genus *Entamoeba*, offering greater insight into the evolution of gene families involved in host–parasite interactions. We focus particularly on the evolution of the cysteine proteases and Gal/GalNAc lectins. We observe that the expansion and contraction of these gene families appears to reflect their rapid evolution and the different host ranges of the various species. We also analyze divergence between the genomes in order to gain evidence of selective pressures acting upon genes within them. We have identified genes under diversifying selective pressures within each species, indicating sequences that are important for survival in the host. Finally, we compare genome-wide diversity levels between and within *E. histolytica* and *E. moshkovskii*. Comparisons of variability between the species and different sequence classes are also made, particularly with a view to establishing the variability of the *E. moshkovskii* genome and whether or not it exists as a species complex (Clark and Diamond 1991, 1997; Heredia et al. 2012).

## Materials and Methods

### Whole-Genome Sequencing of *E. moshkovskii* Strains

Previously described *E. moshkovskii* strains Laredo (ATCC 30042) and FIC (ATCC 30041) are compared alongside two other strains described here for the first time—“15114” and “Snake.” Strain 15114 was received in London from Dr Rashidul Haque (ICDDR, B, Bangladesh), via Dr Bill Petri (UVA), in October 1999 as *E. histolytica*, but was identified as *E. moshkovskii* in August 2000. Strain Snake was received in London from Prof. Jaroslav Kulda (Charles University, Prague, where it had been kept for over 50 years and was thought to be *E. invadens*) in April 2008. Both were adapted to grow axenically by standard methods.

Axenic cultures of *E. moshkovskii* strains Laredo, FIC, 15114, and Snake were grown and maintained in LYI-S-2 media (liver extract, yeast extract, iron, and serum) with 15% adult bovine serum (Clark and Diamond 2002). To culture high cell counts, strains were incubated at room temperature, in darkness, for 7 days. Once at a high density, the cells were centrifuged, washed twice in phosphate-buffered saline solution and lysed with QIAGEN cell lysis buffer, before an adapted version of the previously described CTAB method (Clark and Diamond 1991), as employed by Weedall et al. (2012), with two rounds of the phenol:chloroform:isoamyl alcohol (25:24:1) extraction, was used. The extracted and purified DNA was suspended in nuclease-free water. For strains FIC, 15114, and Snake, 100-bp libraries were pooled and sequenced using the “TruSeq DNA sample prep low throughput protocol” (Illumina), using the in-line control reagent and gel-free method. Libraries were size selected for total fragment lengths between 400 and 600 bp using a Pippin Prep machine (Sage Science) with a 1.5% agarose gel cassette. A 150-bp paired-end (PE) library was similarly generated for the Laredo strain. Assembly of the resulting reads for each strain, and their subsequent application, is described below in the section entitled “Variant Calling and Analysis in *E. histolytica* and *E. moshkovskii*.”

*Entamoeba* *moshkovskii* Laredo was also sequenced using the 454 method in order to generate a de novo assembly. Two single-end fragment libraries, a 3-kb insert PE library and an 8-kb insert PE library were constructed using the manufacturer’s protocols and sequenced using the 454 GS FLX Titanium system (Roche). The Newbler Assembler v2.3 (Margulies et al. 2005) was used to carry out a de novo assembly of the total 3,812,076 generated reads >150 bp using default parameters. The resulting scaffolds, and contigs no smaller than 500 bp, were concatenated to produce an unordered draft assembly.

### Annotation of the *E. moshkovskii* Laredo Genome

A training set of 197 models, including 57 multiexon models, was manually curated for annotation software AUGUSTUS v2.5.5’s training script autoAug (Stanke and Waack 2003). The set was informed using three data sets. Open reading frames 150 amino acids or greater in length were cross-referenced with “hits” generated by entering a 3.5-Mb section of the assembly into a BlastX search (Altschul et al. 1990) against the *E. histolytica* HM-1:IMSS protein set with an exponent value (*E*-value) threshold of 1e-10. Finally, transcriptomic data generated using a previously published protocol (Hon et al. 2013) were used, although default cutoff scores were used with HMMSplicer v0.9.5 (Dimon et al. 2010). AUGUSTUS was then run using default parameters and a set of “hints,” consisting of weighted intron positions inferred from the splice junction data (Bonus = 10, Penalty = 0.7, unweighted values = 1).

Proteins encoded by putative coding sequences (CDSs) in the AUGUSTUS output were entered into a reciprocal BlastP search against the protein set of *E. histolytica* HM-1:IMSS, using default parameters. Predicted sequences with a reciprocal best hit (RBH) were included in the final annotation set. Those without a definite ortholog were included if their total exon length exceeded 350 bp and if they were attributed an AUGUSTUS confidence score of at least 0.75 or they “hit” an *E. histolytica* HM-1:IMSS gene in a one-way BlastP search using an *E*-value threshold of 1e-5.

To add functional annotations to gene models, the *E. moshkovskii* Laredo protein set was entered into reciprocal BlastP searches against the protein sets of *E. histolytica* HM-1:IMSS and *E. dispar* SAW760, using default parameters. Where an *E. moshkovskii* Laredo protein had an RBH against a protein from either of the other species’ sets with a minimum bit-score of 10, the gene by which it was encoded was annotated with the same function as its ortholog. CDSs with RBHs in both *E. histolytica* HM-1:IMSS and *E. dispar* SAW760 were thus functionally annotated twice.

As a measure of completeness, the annotated protein set, along with the protein sets of *E. histolytica* HM-1:IMSS, *E. dispar* SAW760. and *E. invadens* IP-1, was compared with the Benchmarking Universal Single-Copy Orthologs (BUSCO) v3 Eukaryota *obd9* sequence set (Simão et al. 2015) using the BUSCO v3 virtual machine with default settings.

### Reference Strain Data in Other Species

Genomic, CDS and protein sequences, as well as genomic feature files, for *E. histolytica* HM-1:IMSS, *E. dispar* SAW760, and *E. invadens* IP-1 were downloaded from AmoebaDB v2.0 (Aurrecoechea et al. 2010, 2011). Average fold coverage values were acquired from the NCBI Whole Genome Sequence Project pages. The accession numbers for the versions of the three projects used are as follows (with original project accession numbers in parentheses): *E. histolytica* HM-1:IMSS: AAFB02000000 (AAFB00000000); *E. dispar* SAW760: AANV02000000 (AANV00000000); and *E. invadens* IP-1: AANW03000000 (AANW00000000).

### Nonreference Read Data in *E. histolytica*

Existing sequence data for *E. histolytica* strains were used (Gilchrist et al. 2012; Weedall et al. 2012). Strains MS96-3382 and DS4-868, sequenced using Illumina technology, were downloaded from the European Nucleotide Archive (http://www.ebi.ac.uk/ena; last accessed February 2015). Their run accession numbers are SRR368631 and SRR369427, respectively. We used our existing SOLiD-derived read data for *E. histolytica* strains Rahman, 2592100, PVB-M08B, PVB-M08F, HK-9, MS27-5030, MS84-1373 and a cell line derived from the reference strain, HM-1:IMSS-A.

### Defining Orthologs and Gene Families

OrthoMCL v2.0.3 (Chen et al. 2006) was used to identify gene families with orthologs in *E. histolytica* HM-1:IMSS, *E. dispar* SAW760, *E. invadens* IP-1, and *E. moshkovskii* Laredo. Default parameters were used, though an *E*-value threshold of 1e-5 was applied to the All-vs-All BlastP search stage. MySQL served as the relational database. A 50% cutoff value was applied. All proteins from all four species were included in the comparison. MCL was run using a clustering granularity value of 3.0.

### Identification of Orthologs within Virulence Factor Gene Families

*Entamoeba* *histolytica* HM-1:IMSS genes encoding cysteine proteases and Gal/GalNAc lectin subunits were identified using AmoebaDB and NCBI’s Gene Database. Corresponding protein sequences were entered into a TBlastN search against the complete gene sets of *E. histolytica* HM-1:IMSS, *E. dispar* SAW760, *E. invadens* IP-1, and *E. moshkovskii* Laredo to identify orthologs. An *E*-value threshold of 1e-5 and a limit of 50 hits per search were applied to limit the number of poor quality hits and computational expense incurred in analyzing them.

Where 50% or more of a query sequence’s length was cumulatively matched across all hits to a particular reference sequence, that reference sequence and all genes with which OrthoMCL clustered it were added to its respective virulence factor family. Clusters or individual genes present in two families were manually investigated to determine to which family the gene and their cluster should be added. Any identified orthologs lacking functional annotations on AmoebaDB were entered into a BlastP search against the NCBI’s nr database, using default parameters, to subjectively identify any high-quality hits against a member of the virulence factor family to confirm their annotation. In addition to this, any informative or requisite domains or functions were identified using the InterPro and ProtoNet subsections of UniProt. In groups containing noticeably fewer genes in one species, an *E. histolytica* HM-1:IMSS gene within the clade, or an *E. dispar* SAW760 gene in the absence of an *E. histolytica* gene, was entered into a TBLASTX search against the genome of the “missing” species, using default parameters. High-quality hits were determined subjectively, using the *E*-values of known family members. Nonpseudogenous hits were added to their respective virulence factor family.

### Phylogenetic Analyses of Virulence Factor Families

MUSCLE v3.8.31 (Edgar 2004) was used, with default parameters, to align sequences within each family. Bootstrapped maximum likelihood phylograms were generated for each virulence factor family using PHYLIP v3.69 (Felsenstein 1989). Default parameters were used unless otherwise stated. Seqboot was run to generate 1,000 bootstrap pseudoreplicate alignments. Protdist was then run to generate distance matrices for each bootstrap replicate alignment, using the Jones–Taylor–Thornton matrix as well as the gamma distribution of evolution rates among amino acid positions, and proportion of invariant sites if >0, as determined using values calculated by MEGA v5.2.1 using default parameters (Jones et al. 1992; Tamura et al. 2011). Fitch estimated phylogenies with the Fitch–Margoliash criterion for the 1,000 randomized data sets before Consense output bootstrapped trees. To apply branch lengths that represent evolutionary distances to the trees, the first two PHYLIP programs described above were run again, using the same parameters, but for one data set rather than 1,000. Bootstrapped trees were input to Fitch with their respective single data set trees, applying branch lengths to the relationships. Statistical comparisons of branch lengths, representative of evolutionary distances between genes, were manually calculated. Mann–Whitney–Wilcoxon tests (with continuity correction) were performed for each data set using alpha values of 0.05.

In the cysteine protease A subfamily, all incomplete CDSs were entered into a BlastN search against their species’ complete gene set, with an *E*-value threshold of 1e-4. Query sequences and sequences hit by them were accepted as members of the family. Phylogenetic trees for such nucleotide sequence sets were generated using a method similar to the one above but implementing PHYLIP’s DNAdist as opposed to Protdist and using the F84 distance matrix.

### Variant Calling and Analysis in *E. histolytica* and *E. moshkovskii*

Reads from the reference strains (*E. moshkovskii* Laredo reads sequenced for this project; *E. histolytica* HM-1:IMSS-A reads downloaded, as described above) were aligned to the existing assembled reference sequences, downloaded from AmoebaDB v2.0 (Aurrecoechea et al. 2010, 2011), using the Burrows–Wheeler Aligner (BWA) v0.5.9 (Li and Durbin 2009). Default parameters were applied to the “aln” command except in two cases. Firstly, suboptimal alignments were permitted for reads that could be mapped to multiple sites provided that there were no more than ten equally best potential sites. Secondly, maximum edit distances of 4 and 12 were applied to the SOLiD reads and longer Illumina reads, respectively. The “samse” and “sampe” commands were used to align the SOLiD and Illumina reads, respectively, using default parameters. Unmapped and nonuniquely mapped reads were filtered out.

SNPs in the aligned reference strains’ reads were called using the SAMtools v0.1.18 (Li et al. 2009) mpileup command (default parameters were used apart from forcing the output of per-sample read depths) and bcftools view command (default parameters were used except for setting it to output both bases and variants). High-quality SNPs were defined as those that met the following parameters: Phred quality score ≥ 20; read depth ≥ 5 and ≤ 95th percentile of all depths seen in assembly; and farther than 5 bp from a gap, using a window of 30 bp. High-quality homozygous SNPs were inserted in place of their respective original bases within the original reference sequences. The updated reference sequences were then used in place of the original genomes when reads from nonreference strains were mapped, and SNPs called, using the method outlined above.

Total counts of SNPs per gene, excluding pseudogenes and sequences with an incomplete triplet codon, were calculated per strain, distinguishing between synonymous and nonsynonymous SNPs in coding regions and SNPs in noncoding regions. Programs from the phylogenetic analysis using maximum likelihood (PAML) package v4.5 (Yang 1997, 2007) were used to calculate *p*N and *p*S values for each gene relative to each strain’s respective reference strain. A pairwise calculation among all strains within a species would have made the unlikely assumptions that all SNPs were called in each strain and that any base not called as an SNP was definitely the same as in the reference strain. The Probabilistic Alignment Kit (PRANK) v.111130 was run using an empirical codon model with other parameters set to default values, followed by codeml, run using default parameters.

### TMRCA Analysis

To generate time to most recent common ancestor (TMRCA) values for *E. histolytica* and *E. moshkovskii*, all 4-fold degenerate (4D) sites at which only homozygous SNPs were located, and to which reads were mapped at a depth of 35× or greater in all strains of each species, were identified and concatenated. This amounted to 339,091 bases in *E. histolytica* and 641,223 bases in *E. moshkovskii.* The pairwise SNP rates, calculated as fractions of the total number of concatenated 4D sites in *E. histolytica* and *E. moshkovskii*, were used to calculate final “distances”, as well as to visualize, for the first time, the phylogenetic relationships between the strains of *E. moshkovskii*. The generic eukaryotic rate of 2.2 e-9 substitutions per base per annum was considered an acceptable approximation given its use in a similar previous study (Kumar and Subramanian 2002; Neafsey et al. 2012).

PHYLIP v3.69 (Felsenstein 1989) was used to generate neighbor-joining phylograms for nucleotide positions of common 4D sites in *E. moshkovskii* strains and *E. histolytica* strains, using the additive tree model. Default parameters were used unless otherwise stated. Seqboot was run with 1,000 bootstrap replicates. DNAdist was then run using the Jukes–Cantor model, which does not take codon position into account (Jukes and Cantor 1969). Neighbor was subsequently run for the 1,000 data sets, the output of which was processed by Consense. To apply branch lengths that represented evolutionary distances to trees, a distance matrix, consisting of differences between pairs of strains per 4D site, was submitted to Neighbor for a single data set. Branch lengths were manually added to Consense output files.

### Four-Haplotype Test in *E. moshkovskii*

This test was employed to detect meiotic recombination signals between the four tested strains of *E. moshkovskii* in order to determine whether or not they belong to one species or a species complex. One million pairs of high-quality SNPs, defined as nucleotide positions called in every strain and existing as homozygotes in every strain, but varying between them, were randomly sampled. Within groups of 10,000 pairs, proportions of SNP pairs existing as four haplotypes were calculated and the group’s average distance between pairs of sites was calculated. This test was carried out with a previously used Perl script (Weedall et al. 2012).

## Results and Discussion

### Assembly and Annotation of the *E. moshkovskii* Laredo Genome

We sequenced the genome of *E. moshkovskii* Laredo. Of the four DNA libraries sequenced on the 454 GS FLX Titanium system (Roche), the two single-end fragment libraries together yielded 2,211,151 reads (86% > 150 bp). The 3- and 8-kb insert PE libraries generated 743,770 (86% > 150 bp) and 857,155 (90% > 150 bp) reads, respectively. Assembly of the combined total of 3,812,076 reads generated 12,880 contigs. When assembled into scaffolds, 3,352 contigs were included in 1,147 scaffolds. The scaffolds were concatenated, along with 3,460 contigs of at least 500 bp in length, to give a total assembly length of 25,247,493 bp. This is slightly larger than the genomes of the closely related *E. histolytica* and *E. dispar*, but far shorter than that of the more distant *E. invadens* (table 1). However, *E. moshkovskii* is the only *Entamoeba* reference genome that includes contigs not mapped to scaffolds and each of the other genome projects has used different size filtering strategies. The total length of the *E. moshkovskii* genome represented by scaffolds alone is similar to those of the *E. histolytica* and *E. dispar* genomes.

**Table 1:** Statistics Relating to the Genome Assemblies of *Entamoeba histolytica* HM-1:IMSS, *Entamoeba dispar* SAW760, *Entamoeba invadens* IP-1, and *Entamoeba moshkovskii* Laredo

Statistic	*E. histolytica*	*E. dispar*	*E. invadens*	*E. moshkovskii*
Genome length (bp)	20,799,072	22,955,291	40,888,805	25,247,493
GC content (%)	24.20	23.53	29.91	26.54
Non-ACGT (%)	0.31	0.56	0.93	9.94
Number of scaffolds	1,496	3,312	1,149	1,147
N50 of scaffolds (bp)	49,118	27,840	243,235	40,197
Average scaffold size (bp)	13,903	6,931	35,586	19,190
Number of contigs	—	—	—	3,460
Average contig size (bp)	—	—	—	935
Average coverage depth	12.5×**	4.32×*	4×*	82.65×

Note.—Statistics are derived from AmoebaDB v2.0 data, except for asterisked (*) figures, taken from NCBI WGS Projects AANV02 and AANW03; and the double-asterisked (**) figure, taken from Loftus. et al. 2005.

**Fig. 1.**
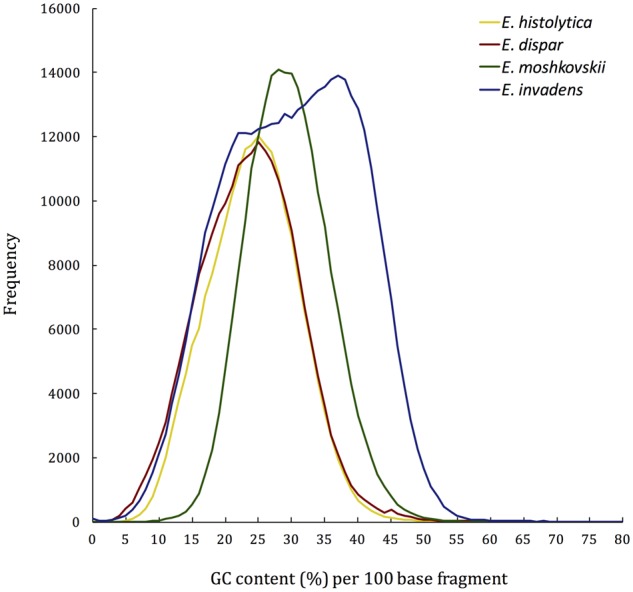
—The range of GC contents in 100 base sections of reference genome assemblies for *Entamoeba histolytica*, *Entamoeba dispar*, *Entamoeba moshkovskii*, and *Entamoeba invadens*. In total, 99.19% of the *E. histolytica* assembly was included, as was 98.49% of the *E. dispar* assembly, 88.75% of the *E. moshkovskii* assembly, and 98.47% of the *E. invadens* assembly.

The average sequence depth for the *E. moshkovskii* assembly is inflated by a relatively small number of contigs and scaffolds with uncommonly high coverage depths (supplementary fig. S1, Supplementary Material online). The modal depth of the assembly was 27× with a mean depth of 82.65×. Exclusion of contigs with coverage depths >2 SD from the mean lowered the average depth to 54.41×. It is likely that such inflated coverage depths are the result of repeat regions in the genome (Sipos et al. 2012; Treangen and Salzberg 2012). The GC content of *E. moshkovskii* is similar to those of the other three species, and the narrow range of GC contents seen across the genome is normally distributed (table 1; fig. 1). Notably, the *E. invadens* genome has an unusual GC distribution compared with the other species, suggesting that different regions of the genome may have different nucleotide biases.

*Entamoeba* *moshkovskii* is predicted to possess 12,449 genes. A total of 216 Eukaryota-lineage BUSCO sequences, including fragmented and duplicated sequences, are represented by this gene content, out of a 303-strong set (table 2). This includes only two fewer complete single-copy BUSCO sequences than in *E. histolytica* and suggests a slightly more complete assembly than is seen for *E. dispar* and *E. invadens*, for which there are a greater number of missing BUSCO sequences. Therefore, we assume that the lack of a complete BUSCO Eukaryota gene set is due to the large evolutionary distance between these protists and the species used to construct the BUSCO gene set. A total of 9,495 genes in the *E. moshkovskii* gene set are predicted to be complete gene models, with the genome containing 2,765 partial genes and 189 pseudogenes. In total, 50.2% of predicted genes are functionally annotated. The final set of gene models, and the concatenated assembly upon which they were based, have been made publicly available as part of AmoebaDB v2.0, released on March 11, 2013. Functional and structural annotations were included in AmoebaDB v4.0.

**Table 2:** Genomic Comparison of *Entamoeba histolytica* HM-1:IMSS, *Entamoeba dispar* SAW760, *Entamoeba invadens* IP-1, and *Entamoeba moshkovskii* Laredo

Statistic	*E. histolytica*	*E. dispar*	*E. invadens*	*E. moshkovskii*
No of CDSs	8,306	8,748	11,549	12,449
Average gene size (bp)	1,280	1,259	1,401	1,230
% Coding DNA	50.12	46.62	38.01	59.04
Average protein size (aa)	418	408	449	399
Average intergenic distance (bp)	1,223	1,365	2,139	798
Proportion of multiexon genes (%)	24.16	30.73	34.48	26.24
Average intron size (bp)	74	81	104	89
Average number of introns per spliced gene	1.27	1.34	1.48	1.31
Number of BUSCO orthologs	220	211	211	216

Note.—Annotation files upon which statistics are based were obtained from AmoebaDB v2.0.

As the manually curated gene set used to train AUGUSTUS was based upon gene models in *E. histolytica*, it is unsurprising that the statistics relating to the *E. moshkovskii* gene set are similar to those seen in *E. histolytica*. *Entamoeba histolytica* possesses the best studied of the genomes here and is the only one to have a manually curated assembly and gene set. This encourages confidence in the gene set predicted for *E. moshkovskii*. However, it does also come with the caveat that mistakes in the *E. histolytica* gene set could be carried into the *E. moshkovskii* set.

### *Entamoeba* Species Show Extensive Expansion and Contraction of Gene Families

**Fig. 2.**
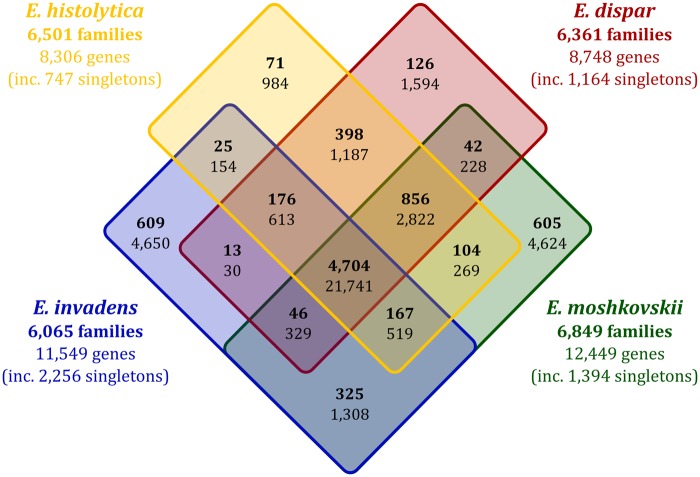
—Venn diagram showing numbers of unique and orthologous genes and families in the genomes of *Entamoeba histolytica, Entamoeba dispar, Entamoeba invadens*, and *Entamoeba moshkovskii*. Numbers are based upon OrthoMCL output. Numbers in bold represent gene families; accompanying numbers in regular font represent the number of genes comprising those gene families.

In order to identify gene families unique to each species, OrthoMCL v2.0.3 was used to cluster sequences in the reference genomes of *E. histolytica*, *E. dispar*, *E. invadens*, and *E. moshkovskii*. A total of 4,704 gene families comprising 21,741 genes were shared by all four species (fig. 2). The number of genes unique to each species positively correlates with the total number of genes in their genomes, as do the number of gene families to which those unique genes belong (table 3).

**Table 3:** Functional Annotations in Genes Unique to *Entamoeba histolytica*, *Entamoeba dispar*, *Entamoeba invadens*, or *Entamoeba moshkovskii*

Number of Families with Function	Number of Genes within Families	Family Function
*E. histolytica*		
6	22	BspA family
4	18	Surface antigen ariel1
2	12	AIG1 family
2	12	Mucins
2	7	Cylicin-2
2	7	Cysteine protease (inc. five pseudogenes)
1	6	Acetyltransferase
*E. dispar*		
1	13	AIG1 family
2	5	Heat shock protein
*E. invadens*		
46	214	Serine/threonine/tyrosine kinase
9	34	Ras family GTPase
2	32	Ribonuclease
8	27	Heat shock protein
1	21	Cylicin
2	21	Myosin
2	19	Glutamine/asparagine-rich protein pqn-25
5	16	Actin
1	15	Thioredoxin
1	12	Profilin
1	11	Capsular polysaccharide phosphotransferase
2	11	DNA double-strand break repair Rad50 ATPase
1	9	Embryonic protein DC-8
3	8	Serine/threonine protein phosphatase
1	8	Tropomyosin alpha-1 chain
2	7	ADP ribosylation factor
2	7	Cysteine protease
1	7	Elongation factor 1-alpha
1	7	Furin
2	6	Actophorin
1	6	Gal/GalNAc lectin light subunit
1	6	Nitrogen fixation protein nifU
1	5	Calcium-binding protein/Caltractin/Centrin-1
2	5	Chaperone Clpb
1	5	DNA repair and recombination protein rad52
1	5	GRIP domain-containing protein RUD3
2	5	Serpin (serine protease inhibitor)
1	5	Vacuolar protein sorting-associated protein
40	753	BspA like family
*E. moshkovskii*		
80	538	Serine/threonine/tyrosine/protein kinase
10	58	Ras family GTPase
5	53	Transposable element/transposase
4	46	Tigger transposable element-derived protein
9	36	Actin
8	26	Heat shock protein
4	17	Leukocyte elastase inhibitor
1	14	Large xylosyl- and glucuronyltransferase 2 isoform X1
2	13	GNAT family *N*-acetyltransferase
1	12	Enhancer binding protein-2
4	11	DNA double-strand break repair Rad50 ATPase
1	10	TonB-dependent siderophore receptor
1	9	Methionine–tRNA ligase
1	9	Tandem lipoprotein
2	8	DEAD/DEAH box helicase
2	8	Reverse transcriptase
1	8	Chaperone
3	7	Methyltransferase (various)
3	7	Cysteine proteinase
1	7	Putative AC transposase
3	6	DNA mismatch repair protein MsH2
2	6	piggyBac transposable element-derived protein
1	6	Polyphosphate:AMP phosphotransferase
1	6	Primary-amine oxidase
1	6	Surface antigen-like protein
1	6	Translation elongation factor
1	6	Type VI secretion system tip protein VgrG
1	6	Site-specific tyrosine recombinase XerC
2	5	Chaperone protein DNAK
1	5	Diaminobutyrate-2-oxoglutarate transaminase
1	5	Response regulator

In the gene set unique to *E. histolytica*, three of the four most prevalent families encode surface proteins. The largest group of genes encodes a 22-gene subset of the *BspA* family (however, other members of the *BspA* family are orthologous to sequences in the other species studied here, demonstrating a role to play in all four species). Totaling 115 sequences in *E. histolytica* alone, the large family lies within one of seven subfamilies containing leucine-rich repeat regions. Multiple BspA-like proteins in *E. histolytica* are located on the plasma membrane of trophozoites (Davis, Zhang, Chen, et al. 2006; Silvestre et al. 2015), and BspA proteins are known to play roles in adhesion to extracellular membranes in both *Bacteroides forsythus* and *Trichomonas vaginalis* (Sharma et al. 1998; Hirt et al. 2002; Noël et al. 2010). It is likely that members of the BspA family are similarly involved in adherence to host cells in *Entamoeba* species. However, the reason for the expanded set of unique *BspA* genes in *E. histolytica* is unclear.

Eighteen *Ariel1* surface antigen family proteins are found in the *E. histolytica*-exclusive gene set, as well as two orthologous serine-rich antigen proteins, whereas there are no Ariel1 genes unique to *E. dispar* and *E. invadens*. The only gene in *E. moshkovskii* annotated as encoding an *Ariel1* surface protein (EMO_091800) is, according to our analysis, unique to *E. moshkovskii*, and forms a cluster with five other unannotated genes. The annotated gene was found, during genome annotation, to potentially possess an incomplete CDS and, as such, its ability to be expressed and the overall function of this gene cluster remains unclear without expression analysis. To a degree, this confirms past research, which noted that the Ariel1 family was present in *E. histolytica* but not in *E. dispar* (Willhoeft U, Buss H, et al. 1999). The family belongs to the same larger family as the *SREHP* protein (Mai and Samuelson 1998), which has been shown to be antigenic (T Zhang et al. 1994); however, the reason for its absence in *E. invadens* and potential lack of functionality in *E. moshkovskii* cannot be determined without further investigation.

In a further comparison of *E. histolytica* and *E. dispar*, 12 members of the AIG1 family are present only in *E. histolytica*, whereas 13 are found only in *E. dispar*. These GTPases, originally isolated in *Arabidopsis thaliana*, are thought to confer resistance to bacterial infections (Reuber and Ausubel 1996; Gilchrist et al. 2006), and have been shown to be more highly expressed in virulent *E. histolytica* cell lines (Biller et al. 2010). The presence of commensal gut microbiota in the environment of trophozoites of both *E. histolytica* and *E. dispar* makes it logical for them to have a large number of genes encoding *AIG1* proteins (49 in total in *E. histolytica*). The different numbers of these genes and the fact that they are undergoing lineage-specific expansions suggest that they are evolving rapidly which is consistent with coevolution with microbial species in the gut.

*Entamoeba* *histolytica* possesses seven species-specific cysteine proteases and three species-specific peroxiredoxins. These genes have roles in invasion and protection from reactive oxygen species, respectively, abilities that are known to be key parts of *E. histolytica’*s pathogenic repertoire (Poole et al. 1997; Davis, Zhang, Guo, et al. 2006; Lidell et al. 2006). However, all of these peroxiredoxin sequences unique to *E. histolytica* are pseudogenes, as are five of the seven cysteine proteases. It is possible that these expansive families are not as significant as once thought, though we consider the cysteine protease families in greater detail below.

There are many more unique genes and families in *E. invadens* than in *E. histolytica* and *E. dispar*. *Entamoeba invadens* possesses genes that encode a number of unique cysteine proteases, thioredoxin proteins, heat shock proteins, and lysozymes. Much of this is likely a direct result of *E. invadens*’ larger gene complement. However, unique expansions of gene families in *E. invadens* may be indicative of a broader host range, an argument strengthened by *E. dispar*—capable of colonizing a range of wild primates (Rivera and Kanbara 1999; Tachibana et al. 2000)—possessing almost twice as many unique genes and families as *E. histolytica*.

Although genes unique to *E. moshkovskii* remain unannotated, given their inherent lack of orthologs, a BLAST search against the NCBI database revealed putative functions for many of them (table 3). As in *E. histolytica*, the most prevalent family (in terms of gene numbers) in these species-specific genes is the BspA family (or genes including a leucine-rich repeat region). Kinases also form a large proportion of the gene families unique to *E. moshkovskii*. It is interesting to note that this species possesses larger unique gene clusters than the other species despite it being more closely related to the human-infective species than *E. invadens*. Notably, its three largest unique gene clusters contain 261, 121, and 110 genes, whereas *E. histolytica’*s largest unique gene cluster contains 13 genes, and *E. dispar’*s and *E. invadens*’ contain 19 and 34 genes, respectively. This large number of hypothetical gene sequences is further evidence of large species-specific gene expansions.

### Comparison of Key Families Involved in Host–Parasite Interactions Suggests Gene Loss in *E. dispar* and Increased Diversity in *E. invadens*

In the course of trying to understand how amoebic lifecycles progress, and occasionally develop into symptomatic disease states, many genes have been identified, including numerous putative virulence factors (Lejeune et al. 2009; Mortimer and Chadee 2010; Wilson et al. 2012). Two major families described above are of particular interest due to their interactions with host cells—the cysteine proteases and Gal/GalNAc lectins. Both families comprised three subfamilies and are heavily implicated in the development of infections, making them exciting targets in the search for potential treatments of amoebiasis. As such, we carried out phylogenetic analyses of these two major gene families and discuss here expansions and reductions within these families in each species in order to assess their importance in the lifecycles of the four *Entamoeba* species.

### Gal/GalNAc Lectins

The Gal/GalNAc lectin heavy subunit allows *Entamoeba* species to adhere to cells by binding to Galactose (Gal) and *N*-acetyl-d-galactosamine (GalNAc) on their membranes (Ravdin and Guerrant 1981). Although there are only two *E. dispar* genes in this family, expansions exist in both *E. histolytica* and *E. moshkovskii*, as well as an expansion in *E. invadens* containing approximately twice as many sequences (supplementary fig. S2*a*, Supplementary Material online). The genes in the expanded *E. invadens* clade are significantly more diverse than the genes in the other two expanded species (based upon branch lengths compared with *E. histolytica*, *P*-value < 0.001; compared with *E. moshkovskii*, *P*-value = 0.045).

The intermediate and light subunits of the Gal/GalNAc lectin offer considerably fewer differences than the heavy subunit (supplementary fig. S2*b* and *c*, Supplementary Material online). The intermediate subunit group contains an *E. invadens* expansion only, raising the number of *E. invadens* genes above the number of genes seen in the other species (mean branch length: 2.962247; *s* = 1.610896). The light subunit family, meanwhile, contains two *E. invadens* expansions and a smaller expansion in both *E. dispar* and *E. histolytica*, but no expansion in *E. moshkovskii.* Again, the *E. invadens* expansions are more variable than those of *E. histolytica* (*P*-value = 0.002) and *E. dispar* (*P*-value = 0.002).

Interestingly, all of the *E. invadens* genes encoding Gal/GalNAc lectin heavy subunits have orthologs in the other three species. Given that *E. invadens* is capable of causing amoebic infections in a variety of reptilian hosts, one can theorize that the variable Gal/GalNAc lectin heavy subunits are a key family in allowing *E. invadens* to do so. However, regardless of target host, Gal/GalNAc lectin heavy subunit proteins share enough similarities to be considered orthologous.

Conversely, there is a relative lack of heavy subunit sequences in *E. dispar*. As was suggested in the case of the BspA family, a paucity of proteins required for adherence to host cells may explain why symptomatic disease is seen so infrequently in this species (Diamond and Clark 1993; Ximénez et al. 2010). A reduced complement of genes encoding proteins involved in host–parasite adherence suggests a diminished requirement for this type of protein, at least relative to the other species studied here. This could be a crucial characteristic of *E. dispar* that distinguishes it from its relatives. Furthermore, the relative lack of variability in the light and intermediate lectin subunits, when compared with the heavy subunit subfamily, suggests that the smaller subunits are less crucial to the success of amoebic infections than the heavy subunit.

### Cysteine Proteases

The cysteine proteases can be divided into three subfamilies—A, B, and C. In subfamily A (supplementary fig. S2*d*, Supplementary Material online), there are more *E. invadens* genes than there are genes from the other species, and a notably lower number of *E. dispar* sequences. The higher number of *E. invadens* genes is due to a lineage-specific expansion (mean branch length: 0.814269; *s* = 0.266459). A pseudogenous *E. dispar* sequence, meanwhile, lies in a region syntenic to *E. histolytica*’s *CP-A5* (Willhoeft U, Hamann L et al. 1999). This gene has been shown to be important in the virulence phenotype of *E. histolytica* (Bruchhaus et al. 2003). There are no other *E. dispar* pseudogenes, whereas there are nine *E. histolytica* pseudogenes.

In subfamily B (supplementary fig. S2*e*, Supplementary Material online), *E. dispar* possesses considerably fewer genes than the other three species, as it is the only species whose genes have not been subject to expansion. The *E. moshkovskii* gene expansion is significantly more diverse (*P*-value < 0.001) but relatively closely related to the *E. histolytica* expansion, being part of the same clade. Conversely, the expanded *E. invadens* genes are more varied than both the *E. moshkovskii* sequences (*P*-value < 0.001) and the *E. histolytica* sequences (*P*-value < 0.001) and have expanded in an independent event. As was seen in subfamily A, *E. invadens* appears to possess a larger, more variable set of cysteine proteases than the other three species. Comparatively, in subfamily C (supplementary fig. S2*f*, Supplementary Material online), there are fewer *E. invadens* genes than there are of the other three species. This is due to a large clade consisting mostly of very similar sequences across those three species (mean branch length: 0.266321; *s* = 0.351707).

The relative paucity of *E. dispar* cysteine protease sequences (33 CDSs, compared with 42 in *E. histolytica*, 51 in *E. invadens*, and 46 in *E. moshkovskii*) suggests a diminished requirement for these proteins, as was the case with the Gal/GalNAc lectins, above. Taken alongside the fact that *E. dispar* is the only one of the four species to have a pseudogenized ortholog of the important *CP-A5* gene (Bruchhaus et al. 1996; Ankri et al. 1999), it appears that *E. dispar* has experienced a general reduction in a family of genes which are known to be involved in host invasion as well as having generalized proteolytic abilities. This reduction is likely to be at least partly responsible for its apparently reduced impact upon host cells, which has long been recognized in the literature (Diamond and Clark 1993). Conversely, the large number of cysteine proteases in *E. moshkovskii* is consistent with studies that suggest *E. moshkovskii* is capable of causing symptomatic infection in humans (Fotedar et al. 2008; Shimokawa et al. 2012). *Entamoebainvadens* also appears to require a variety of cysteine proteases further supporting the theory that *E. invadens* requires a greater diversity of virulence factors to allow it to effectively parasitize its wide range of hosts.

### Intraspecies Diversity in *E. moshkovskii* Relative to *E. histolytica*

We investigated genomic diversity between strains of *E. moshkovskii* and *E. histolytica*. This required nonreference strains, which were unavailable for *E. dispar* and *E. invadens*, so these species could not be compared here. Reference strains were resequenced using Illumina sequencing, and reads were mapped to their respective genomes (supplementary table S1, Supplementary Material online). Existing bases at high-quality homozygous positions were replaced with the newly called nucleotides to generate updated and improved reference sequences. Reads from nonreference strains were mapped to these updated reference genomes and SNPs were called within them (table 4). Every strain except *E. histolytica* strain PVF was sequenced and mapped to a coverage depth higher than 35×, the average necessary to reliably detect 95% of SNPs in a genetic sequence (Ajay et al. 2011; Sims et al. 2014). The nonreference *E. moshkovskii* strains mapped to coverage depths equivalent to, or higher than, those achieved with the *E. histolytica* strains sequenced on the Illumina platform.

**Table 4:** Mapping and Coverage Statistics for Each Strain Studied in This Project

Strain	Country of Origin	Sequencing Platform	Year of Isolation	Average Coverage Depth (*x*)	No of Mapped Reads	Coverage of Ref. (%)
*Entamoeba histolytica*						
HM-1:IMSS-A^a,b^	Mexico	SOLiD 4	1967	43.53	13,743,197	61.03
2592100^c^	Bangladesh	SOLiD 4	2005	41.50	13,618,188	68.83
HK-9^d^	Korea	SOLiD 4	1951	57.41	21,217,510	71.86
PVBM08B^c^	Italy	SOLiD 4	2007	50.02	17,688,152	70.88
PVBM08F^c^	Italy	SOLiD 4	2007	29.61	8,506,016	71.88
Rahman^e^	UK	SOLiD 4	1964	49.43	19,534,522	67.78
MS27-5030^c^	Bangladesh	SOLiD 4	2006	59.97	20,419,790	63.27
MS84-1373^c^	Bangladesh	SOLiD 4	2006	63.01	21,499,758	69.57
MS96-3382^f^	Bangladesh	Illumina GA II	2007	114.03	20,527,917	89.00
DS4-868^g^	Bangladesh	Illumina GA II	2006	72.15	13,361,613	88.36
*Entamoeba moshkovskii*						
Laredo^h^	America	Illumina MiSeq	1956	97.61	8,833,683	89.91
FIC^i^	Canada	Illumina MiSeq	1959	162.27	19,750,749	61.58
Snake	France*	Illumina MiSeq	1948*	209.10	25,655,106	76.96
15114	Bangladesh	Illumina MiSeq	1999	265.55	35,292,777	85.24

Note.— Gray rows represent reference strains, reads from which were mapped to their existing respective reference genome. Positions at which high-quality homozygous SNP calls were made in the reads were replaced in the original reference sequence. All other strains were mapped to the updated versions of their respective reference strains. Underlined sections of strain names represent the shortened versions of the names that will be used henceforth. References: a) Biller et al. (2009); b) Biller et al. (2010); c) Weedall et al. (2012); d) Ungar et al. (1985); e) Diamond and Clark (1993); f) Gilchrist et al. (2012); g) Ali et al. (2007); h) Dreyer (1961); i) Meerovitch (1958).

* Sent from Institut Pasteur, Paris to Charles University, Prague in 1948. Institut Pasteur has no record of origin (Clark G, London School of Hygiene and Tropical Medicine, personal communication).

Pairwise SNP rates, including heterozygous and homozygous SNPs, were calculated across all genotype quality scores for each nonreference strain relative to its respective reference genome as a measure of divergence (supplementary fig. S3, Supplementary Material online). The average divergence of all *E. moshkovskii* strains from the reference was greater than that demonstrated by *E. histolytica* strains compared with the HM-1:IMSS strain (Wilcoxon signed-rank test: *P*-value < 0.01), a difference apparently independent of genotype quality. Within *E. moshkovskii*, the three nonreference strains’ divergence from Laredo suggested that the only human-infective nonreference strain—15114—is the least divergent from the similarly human-infective Laredo (compared with Snake: *P*-value < 0.01; compared with FIC: *P*-value < 0.01). The sewage-derived strain FIC is significantly more divergent than both host-derived 15114 and Snake (compared with Snake: *P*-value < 0.01). Taken together, these relationships suggest lineages diverging to facilitate, or as a result of, parasitic abilities.

### *Entamoeba moshkovskii* Strains Display Greater Divergence from Their Reference Strain than *E. histolytica* across All Sequence Classes

**Fig. 3.**
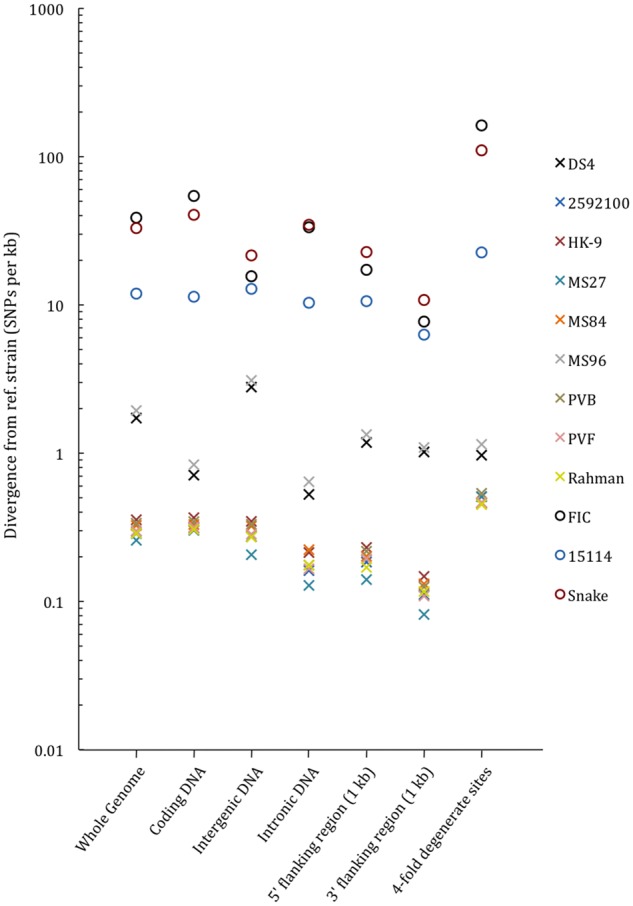
—Divergence of *Entamoeba histolytica* and *Entamoeba moshkovskii* strains, relative to their reference strains (HM-1:IMSS and Laredo, respectively), within different sequence classes. Circles represent *E. moshkovskii* strains, and crosses represent *E. histolytica* strains. SNPs occurring in regions classified as both flanking regions and coding regions were considered to occur in coding regions only. Rates are relative to sites within their respective sequence classes.

SNP rates in a range of sequence classes were studied in more detail (fig. 3). Both homozygous and heterozygous SNPs were included in this analysis. Mann–Whitney statistical tests were used to compare the average divergence between sequence classes between the species. An alpha level of 0.05 was used for all tests. Statistically significant differences in divergence were found between the *E. histolytica* and *E. moshkovskii* strains in all sequence classes (for 4D sites and intronic regions, *P*-value = 0.02; for all other classes, *P*-value < 0.01). This confirms that the greater divergence seen in *E. moshkovskii* is ubiquitous across the genome.

Overall, diversity seen between the four strains of *E. moshkovskii* was 200 times greater than that seen between ten strains of *E. histolytica* (supplementary fig. S4, Supplementary Material online). This higher diversity was seen, to varying degrees, ubiquitously across all sequence classes, including noncoding DNA. Noncoding DNA contains a wealth of regulatory elements involved in the control of such important processes as DNA replication and gene expression (Ludwig 2002; Bracha et al. 2003, 2006; Nelson et al. 2004; Anbar et al. 2005; Wilusz et al. 2009; Mar-Aguilar et al. 2013); therefore, these differences will likely result in important phenotypic differences.

Within *E. moshkovskii* and *E. histolytica*, occurrences of polymorphisms in coding regions were compared with those in a variety of classes of noncoding regions. There were no significant differences in divergence seen in coding regions and those values recorded for the noncoding regions in *E. moshkovskii*. Conversely, coding regions of *E. histolytica* genomes were, overall, significantly more divergent than intronic regions (*t* = 15.0988, df = 7, *P*-value = 1.34e-6) and 3′-flanking regions (*t* = 2.5806, df = 7, *P*-value = 0.036), suggesting that polymorphisms occur at different rates in these regions of *E. histolytica*. This could not be proven convincingly in *E. moshkovskii*, possibly implying a greater importance of some non-CDS in *E. moshkovskii*.

As intergenic regions in *Entamoeba* genomes are very short, it may be that they are densely packed with regulatory regions. Our findings contradict a previous study that focused on individual genes and associated noncoding regions in *E. histolytica* and which suggested that the latter were more divergent than coding regions due to their being under less selective pressure (Bhattacharya et al. 2005). However, it is likely that the difference between the two conclusions is because the analyses featured here were performed across the entire genome, as opposed to selected regions, and so are based upon more data.

The 5′- and 3′-flanking regions of a sequence typically contain promoter and enhancer regions, to which transcription factors sometimes bind (Riethoven 2010). SNPs in 5′-flanking regions are known to affect regulation and expression levels (Hayashi et al. 1991; Marcos-Carcavilla et al. 2010; Peñaloza et al. 2013). The effects of promoter-based SNPs on stress resistance have previously been reported, so it is conceivable that SNPs in 5′- and 3′-flanking regions could facilitate, as an example, survival outside of a human host (Sun et al. 2007). However, it is likely that the differences in diversity between *E. histolytica* and *E. moshkovskii* are due to greater divergence within the latter, as opposed to selective pressures acting upon particular regions of the genome such as this argument would require.

### TMRCA Analysis Suggests a Recent Origin for *E. histolytica*

**Fig. 4.**
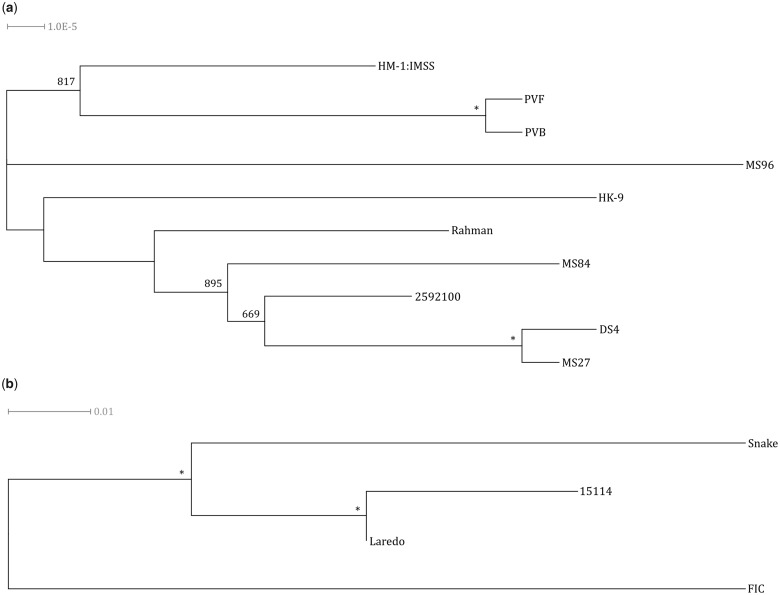
—Phylogenies of (*a*) *Entamoeba histolytica* and (*b*) *Entamoeba moshkovskii* strains based upon diversity in 4D synonymous sites. The trees were generated using a Neighbor-Joining method and are unrooted. Asterisks at all branching points indicate bootstrapping values of 1,000 out of 1,000. Branching points’ missing values were not supported by bootstrapping.

As stated above, divergence across strains’ 4D sites was greater in *E. moshkovskii* than in *E. histolytica* (fig. 3). Such sites have long been thought to be under neutral selective pressure, given that mutations in them do not affect the amino acid that their triplet encodes (Kimura 1968; King and Jukes 1969). As such, they provide an opportunity to evaluate the overall differences in diversity between species without the added complication of selective pressures influencing results. With this in mind, the 4D sites present in *E. histolytica* and *E. moshkovskii* that were sequenced to depths of 35× or greater in all strains of a species (339,091 and 641,223 bases, respectively) were employed to approximate, for each species, the age of the most recent ancestor shared by the tested strains to further evaluate relatedness between strains of the species. The TMRCA for *E. histolytica* is estimated to be 165,000 years, whereas the TMRCA for the *E. moshkovskii* strains is ∼81,590,000 years. This suggests an origin of *E. histolytica* that is concurrent with the emergence of modern humans, whereas it suggests that *E. moshkovskii* is much more ancient. Indeed, it is likely that this ancestral species, predating as it does mammals, has diverged many times, with descendants coevolving with mammalian hosts through myriad lineages to parasitize the wide range of hosts we see *Entamoeba* species infecting today, whereas *E. moshkovskii* remains recognizable as a contemporary organism. This theory is not without precedent, having been suggested previously concerning the infection by basal coccidians of ancestral vertebrates such as elasmobranchs (Xavier et al. 2018). The subsequent coevolution and divergence of these parasites with the dawn of their higher vertebrate hosts is thought to have produced the genus *Toxoplasma* among others (Rosenthal et al. 2016). Phylogenetic analyses of both *E. histolytica* and *E. moshkovskii* demonstrated that observed variation between strains was not a result of significantly more distant reference strains (fig. 4). It should, however, be acknowledged that the assumed mutation rate is not specific to the *Entamoeba* species so the accuracy of these TMRCA values cannot be validated.

### Identification of Genes under Diversifying Selection in *E. moshkovskii* and *E. histolytica*

In order to identify genes under diversifying selective pressures within each species and thereby identify genes that are under positive selection from the host, ratios of *p*N to *p*S values (*p*N/*p*S) were calculated for each CDS in each strain of *E. histolytica* and *E. moshkovskii* relative to their respective reference genomes. Numerous variations on such comparisons of synonymous and nonsynonymous substitution rates have led to the identification of many CDSs under positive selection in a wide range of species, as summarized by Yang and Bielawski (2000). The concept’s history of power and reliability in such cases, and the relatively simplicity of its calculation, made it an ideal choice for detection of selection in *Entamoeba* species. Heterozygous SNPs were omitted as calculation of the impact they have upon a sequence’s *p*N/*p*S ratio would have been impractical. Annotations were taken from orthologous sequences where none was available for sequences themselves.

In *E. moshkovskii*, the majority of genes identified as being under diversifying selection lacked annotations or known domains (supplementary table S2, Supplementary Material online). A relatively large number of BspA family members were found to be under diversifying selective pressures in all three strains, suggesting a species-wide function. In addition to the BspA family proteins, all three *E. moshkovskii* strains were found to possess genes with similar housekeeping functions in the form of protein kinases, DNA repair proteins, and Ras family GTPases. Although there are numerous *Entamoeba* proteins involved in cell adherence, including the *Ariel1* surface antigen seen to be under diversifying selection in *E. moshkovskii* strain 15114, the *BspA* family is the only adherence-related family seen to be under such pressures in all three strains. It would be of great interest to study how crucial the BspA family is in enabling adherence to host cells in *E. moshkovskii*.

*p*N/*p*S ratios indicating diversifying selective pressures acting upon genes were present in eight of the nine nonreference *E. histolytica* strains, with only HK-9 appearing to lack sequences under such pressures (supplementary table S3, Supplementary Material online). However, the numbers recorded in each strain were, compared with counts in *E. moshkovskii*, very low, with only MS96 featuring more than five such diversified genes. Of those genes identified as being under diversifying selection, one can see that the majority are unannotated, but, as in the *E. moshkovskii* strains, there appear *BspA* family proteins in strain MS96 as well as AIG1 family members in MS84 and MS96, and serine/threonine protein kinases in the Illumina-sequenced strains.

The comparatively low numbers of genes under diversifying selection in *E. histolytica* are likely the result of a combination of factors. Firstly, every *E. histolytica* strain excluding DS4 and MS96 was sequenced to a relatively low depth, as a result of differing sequence technologies. As such, fewer SNPs were likely to have been detected, thus profoundly affecting the calculation of *p*N/*p*S ratios. However, even taking this into account, we do see very few genes in MS96 and DS4 under diversifying selective pressures compared with strains of *E. moshkovskii*. This supports our findings that *E. histolytica* is significantly less functionally diverse than *E. moshkovskii*. Secondly, *p*N/*p*S ratios can only be accurately calculated where a sequence contains both synonymous and nonsynonymous SNPs. It was likely that many genes containing only nonsynonymous SNPs, which would still certainly be classed as being under diversifying selection, would have been omitted. This would, of course, have also affected the *p*N/*p*S ratios in *E. moshkovskii*.

### Weak Signals of Meiotic Recombination in *E. moshkovskii* Suggest It Is a Species Complex

The high level of genetic diversity and ancient estimate of the TMRCA indicates that *E. moshkovskii* may in fact not be a true species but a species complex, a group of genetically isolated lineages brought together under a single species name. This has been previously suggested by Clark and Diamond (1997) using riboprinting. The four-haplotype test was used to check for evidence of meiotic recombination between the four *E. moshkovskii* strains. According to the infinite sites model of evolution, individual nucleotide positions can only mutate once, meaning that the maximum possible number of haplotypes between two physically linked sites is three, unless recombination between genomes is possible. Furthermore, recombination is more likely to occur between sites the greater the distance between them. As such, the occurrence of four haplotypes within a species, combined with a greater prevalence of such haplotypes over greater genomic distances act as reliable indicators of meiotic recombination. Evidence of meiotic recombination has previously been reported in *E. histolytica*, demonstrating that it can occur in members of the genus *Entamoeba* (Weedall et al. 2012). A Spearman’s correlation coefficient was applied to test whether, in the *E. moshkovskii* strains tested here, there was any significant correlation between the proportions of physically linked SNP pairs that exist as four haplotypes and the distance between members of those pairs (supplementary fig. S5, Supplementary Material online). There was no significant correlation, meaning that four-haplotype SNP pairs are not more prevalent over greater distances as would be expected if there was a strong signal of meiotic recombination between these four strains. Four distinct haplotypes were observed in *E. moshkovskii*, although at a much lower frequency than in *E. histolytica*, and we assume these are due to ancient recombination or where the infinite sites model does not hold.

Although this suggests that the four strains of *E. moshkovskii* studied here do not belong to the same species, this result necessitates two important caveats. Firstly, conclusions drawn from these data do not necessarily extend beyond the strains featured and our results do not preclude the probable occurrence of recombination in any of the subspecies that make up the *E. moshkovskii* complex. Secondly, given that the strains compared to identify genes under selective pressures in *E. moshkovskii* have been shown to not all be of the same species, such identified genes may have been selected for in an ancestral population, rather than currently undergoing selection. Finer resolution of these cases will no doubt be provided by future investigations into the species complex.

## Conclusions

Through sequencing the genomes of four strains of *E. moshkovskii*, including the generation of an annotated reference genome, we have performed a comparative analysis of *E. moshkovskii* against its relatives *E. histolytica*, *E. invadens*, and *E. dispar*. The genome of *E. moshkovskii* reference strain Laredo contains 12,449 predicted CDSs. Although many of these are incomplete, the assembly and annotation comprise a good-quality first draft of the genome. We have also undertaken a preliminary analysis of genomic diversity in *E. moshkovskii* by sequencing four isolates using short-read sequencing technology. This, combined with existing genomic resources for *E. histolytica, E. dispar*, and *E. invadens*, has enabled a detailed analysis of genomic diversity and gene family evolution in the different species.

Surface-bound proteins are implicated in playing a major role in the development of amoebiasis. The pathogenic *E. histolytica* possesses a large number of unique surface proteins, which contrasts starkly with the nonpathogenic *E. dispar*. This study also suggests that other surface-bound proteins might play roles similar in importance to the Gal/GalNAc lectins with regards to enabling pathogenic infections in the genus. Furthermore, *E. invadens* was found to possess a greater number of genes in the Gal/GalNAc lectin heavy subunit subfamily and the cysteine protease subfamilies A and B than the other three species studied. The genes comprising the expansions in these families were also often significantly more variable than those genes seen in the other *Entamoeba* species. It is reasonable to conclude that a proportion of the enlarged gene set seen in *E. invadens* (relative to *E. histolytica* and *E. dispar*) consists of genes required to facilitate the amoeba’s polyxenous lifestyle. This argument could be extended to *E. dispar* relative to *E. histolytica*; however, the low numbers of surface proteins seen in *E. dispar* are also seen in its cysteine protease virulence factor gene families.

Overall, the genomes of the studied *E. moshkovskii* strains were found to be more diverse than those of the *E. histolytica* strains, with the former species ∼200 times as diverse as the latter. This greater diversity was found to be the case across multiple sequence classes, demonstrating that it is not restricted to individual regions of the genome. Furthermore, *E. moshkovskii* was found to have diverged from its strains’ most recent common ancestor nearly 500 times longer ago than *E. histolytica’*s strains did from theirs. It is likely, therefore, that the reason for the greater diversity within *E. moshkovskii* is that its genome has accrued mutations over a longer period of time than that of *E. histolytica*, thus suggesting that genetic diversity is very low in *E. histolytica*.

Our data indicate that *E. moshkovskii* strains are probably not the same species. This is important for understanding its relationship to human infection. It may be that only one of these sequence types can be infective and, therefore, to understand the epidemiology of this emerging disease we need to develop better diagnostics that can differentiate between the different sequence types. Also, if there are pathogenic and nonpathogenic types of *E. moshkovskii*, they could act as a useful system for studying the emergence of pathogenicity. Our attempts to identify gene families of importance in survival of the varied lifestyles exhibited by *E. histolytica* and *E. moshkovskii* identified the BspA family as a putatively important family in members of the *E. moshkovskii* complex. Given the family’s role in *E. histolytica*, and absence from the genome of *E. dispar*, it is possible that members of the *E. moshkovskii* species complex may be capable of causing disease in human hosts.

## Supplementary Material

Supplementary data are available at *Genome Biology and Evolution* online.

## Supplementary Material

Supplementary Data

## Notes

**Data deposition:** GenBank assembly accession: GCA_002914575.1: BioSample: SAMN07660612: BioProject: PRJNA407662: WGS Project: NWBR01 (GenBank: NWBR00000000.1).
