# Supplementary material for: Genetic Diversity and Gene Family Expansions in Members of the Genus Entamoeba
Source: Genome Biol Evol. 2019 Jan 21;11(3):688–705. doi: 10.1093/gbe/evz009 (PMC6414313; doi:10.1093/gbe/evz009)
Supplement: Supplementary Data [file evz009_supp.zip › All_Supplementary_figures_revised.pdf]

Fig S1. Frequencies of mean read depths within each scaffold/contig in the *Entamoeba moshkovskii* Laredo genome. The highest fold coverage recorded was 7730.04x.

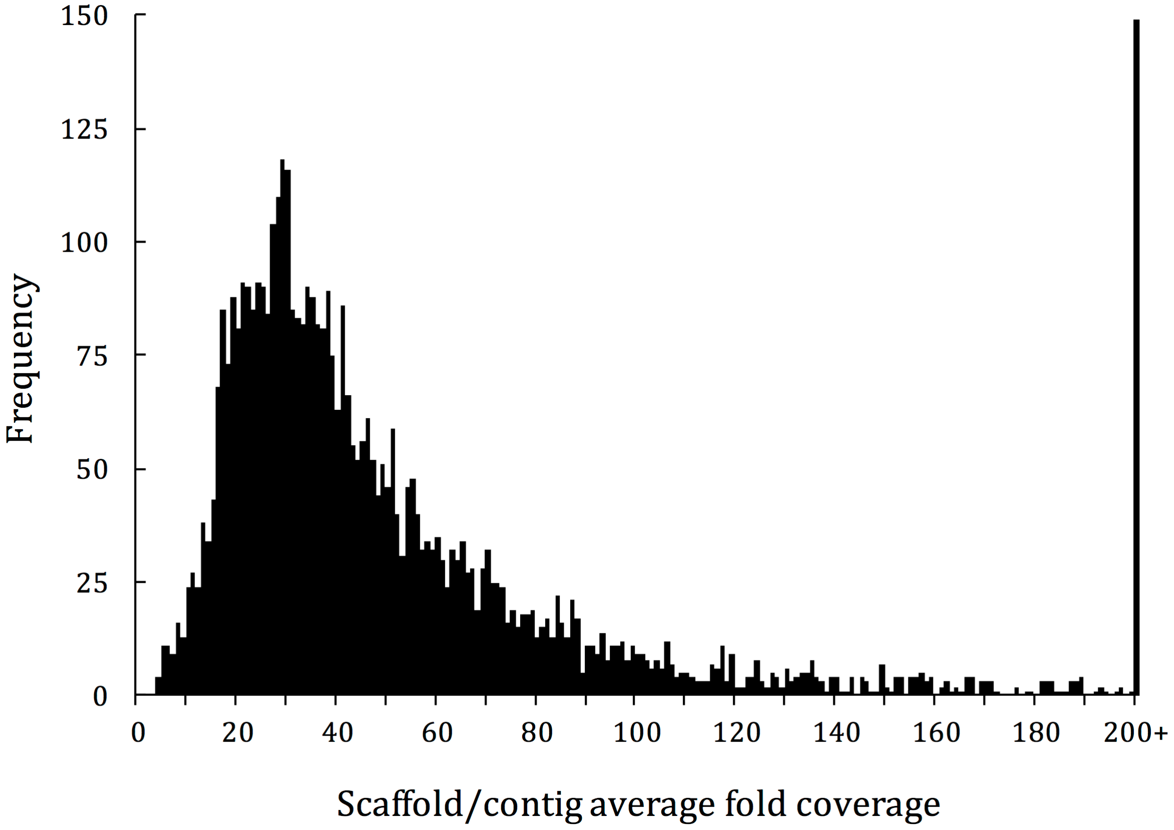

Fig S2. Phylograms of *Entamoeba* gene families directly involved in virulence that demonstrate differential expansions and reductions across species. Red boxes highlight clades in which pseudogenes were identified. They are linked to red boxes showing the same clades when phylogeny was calculated using nucleotide sequences, including the pseudogenes. Scale bars in red boxes represent nucleotide phylograms. All phylograms are midpoint rooted. Bootstrapping was performed for 1,000 replicates. Bootstrap values of 1,000 are represented by asterisks (*). Bootstrap values below 400 are not shown. a) Heavy Gal/GalNAc lectin subunits; b) Intermediate Gal/GalNAc lectin subunits; c) Light Gal/GalNAc lectin subunits; d) Cysteine protease Family A; e) Cysteine protease Family B; f) Cysteine protease Family C.

a)

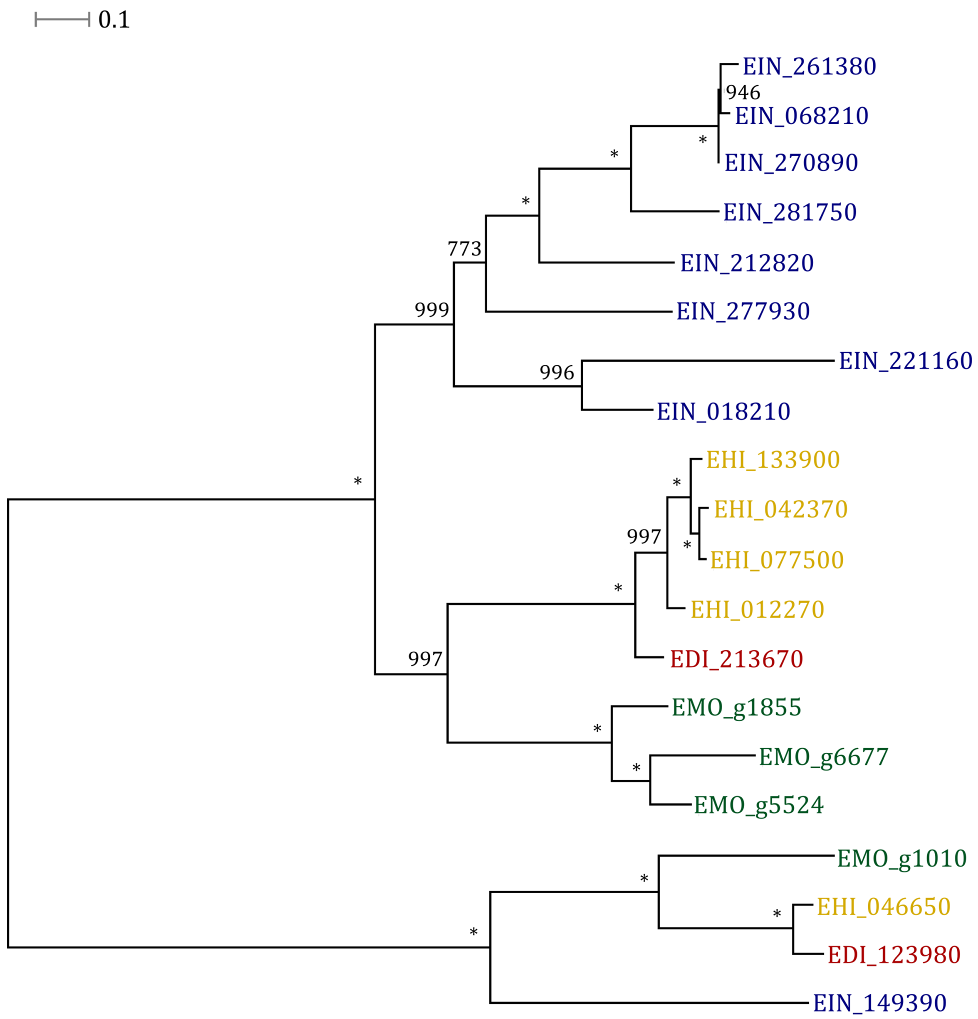

b)

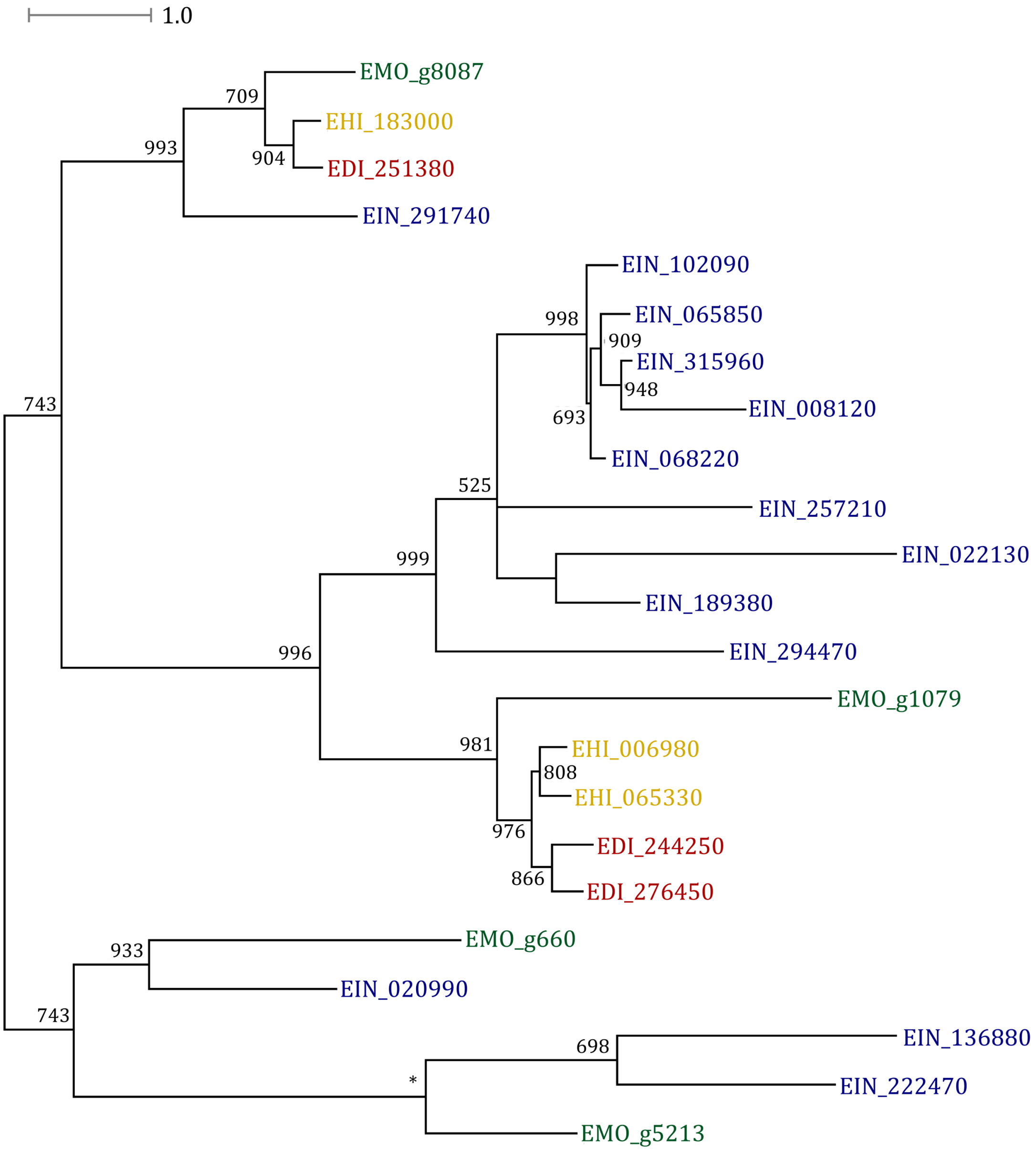
1.0
EMO_g8087
709
EHI_183000
993
904
EDI_251380
EIN_291740
EIN_102090
998
EIN_065850
909
EIN_315960
948
EIN_008120
693
743
EIN_068220
525
EIN_257210
999
EIN_022130
EIN_189380
996
EIN_294470
EMO_g1079
981
EHI_006980
808
EHI_065330
976
EDI_244250
866
EDI_276450
933
EMO_g660
EIN_020990
743
EIN_136880
698
*
EIN_222470
EMO_g5213

c)

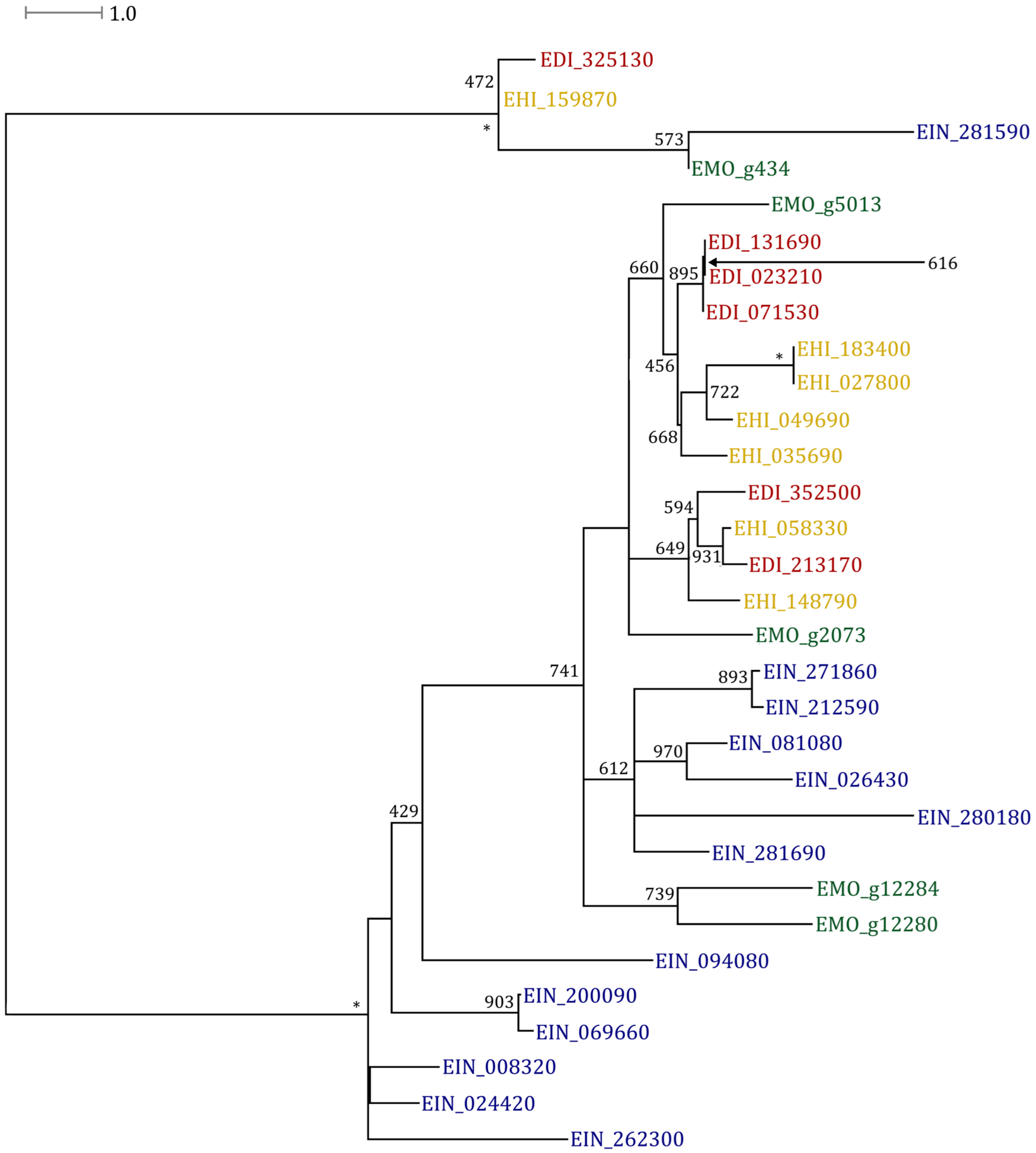
1.0
EDI_325130
472
EHI_159870
*
573
EIN_281590
EMO_g434
EMO_g5013
EDI_131690
616
660
895
EDI_023210
EDI_071530
EHI_183400
*
456
EHI_027800
722
EHI_049690
668
EHI_035690
EDI_352500
594
EHI_058330
649
931
EDI_213170
EHI_148790
EMO_g2073
741
893
EIN_271860
EIN_212590
EIN_081080
970
612
EIN_026430
EIN_280180
EIN_281690
429
739
EMO_g12284
EMO_g12280
EIN_094080
903
EIN_200090
*
EIN_069660
EIN_008320
EIN_024420
EIN_262300

d)

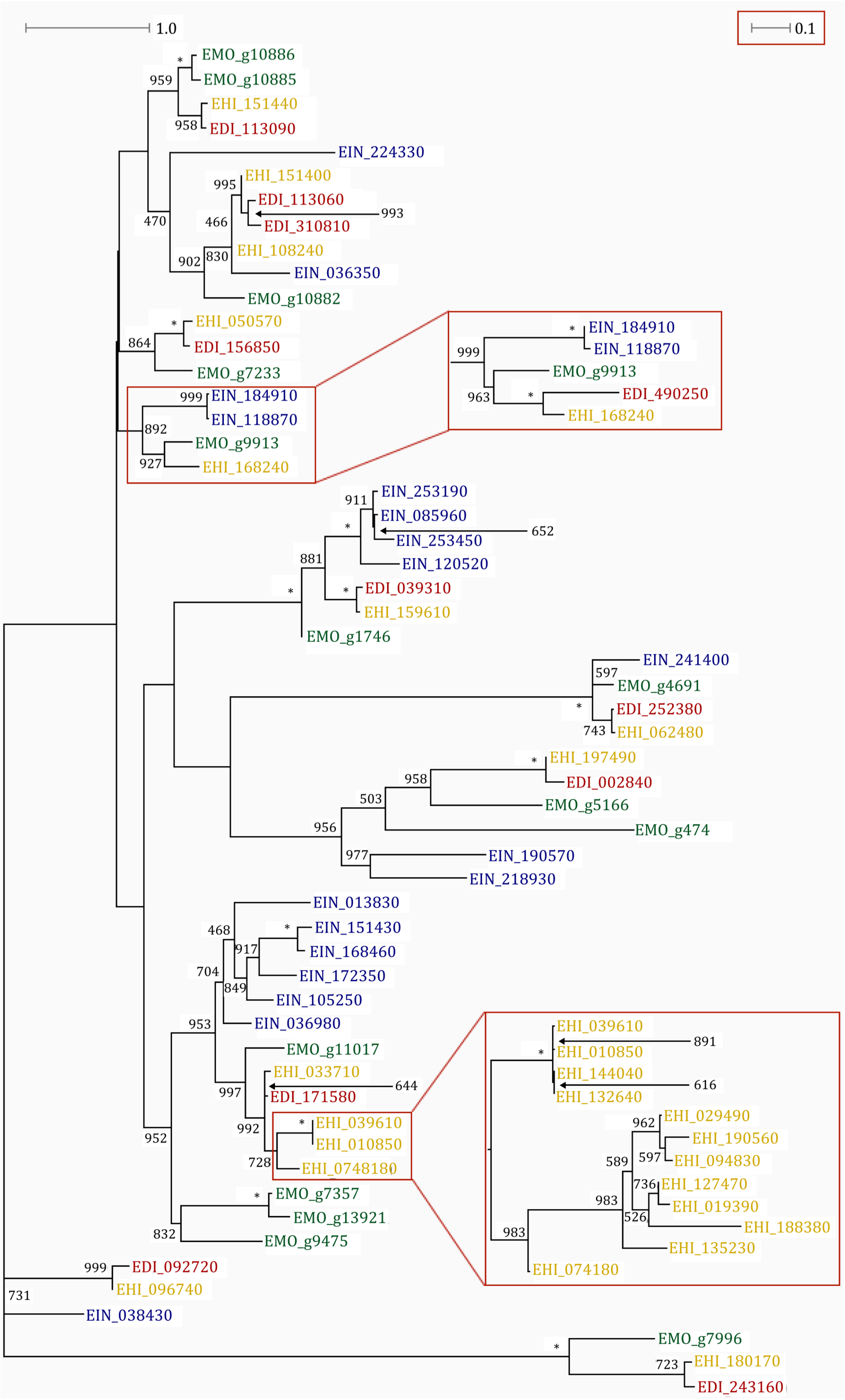

1.0
0.1
EMO_g10886
EMO_g10885
EHI_151440
EDI_113090
EIN_224330
EHI_151400
EDI_113060
993
EDI_310810
EHI_108240
EIN_036350
EMO_g10882
EHI_050570
EDI_156850
EMO_g7233
EIN_184910
EIN_118870
EMO_g9913
EHI_168240
EIN_184910
EIN_118870
EMO_g9913
EDI_490250
EHI_168240
EIN_253190
EIN_085960
652
EIN_253450
EIN_120520
EDI_039310
EHI_159610
EMO_g1746
EIN_241400
EMO_g4691
EDI_252380
EHI_062480
EHI_197490
EDI_002840
EMO_g5166
EMO_g474
EIN_190570
EIN_218930
EIN_013830
EIN_151430
EIN_168460
EIN_172350
EIN_105250
EIN_036980
EMO_g11017
EHI_033710
644
EDI_171580
EHI_039610
EHI_010850
EHI_0748180
EHI_039610
891
EHI_010850
EHI_144040
616
EHI_132640
EHI_029490
EHI_190560
EHI_094830
EHI_127470
EHI_019390
EHI_188380
EHI_135230
EHI_074180
EMO_g7357
EMO_g13921
EMO_g9475
EDI_092720
EHI_096740
EIN_038430
EMO_g7996
EHI_180170
EDI_243160

e)

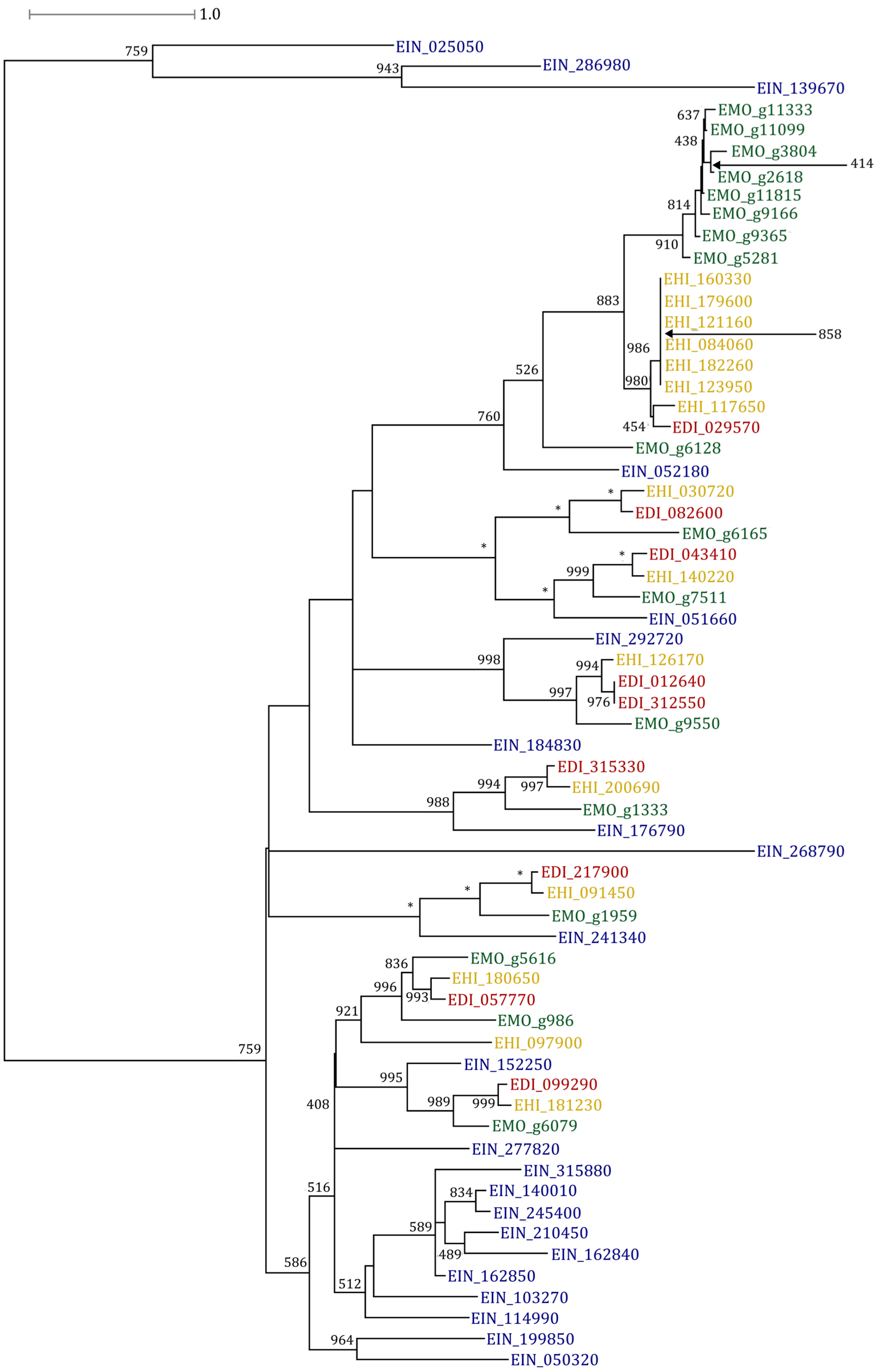
1.0
759
EIN_025050
943
EIN_286980
EIN_139670
637
EMO_g11333
438
EMO_g11099
EMO_g3804
414
EMO_g2618
814
EMO_g11815
EMO_g9166
910
EMO_g9365
EMO_g5281
883
EHI_160330
EHI_179600
EHI_121160
858
986
EHI_084060
526
EHI_182260
980
EHI_123950
EHI_117650
760
454
EDI_029570
EMO_g6128
EIN_052180
*
EHI_030720
*
EDI_082600
EMO_g6165
*
*
EDI_043410
999
EHI_140220
*
EMO_g7511
EIN_051660
EIN_292720
998
994
EHI_126170
EDI_012640
997
976
EDI_312550
EMO_g9550
EIN_184830
EDI_315330
994
997
EHI_200690
988
EMO_g1333
EIN_176790
EIN_268790
*
EDI_217900
*
EHI_091450
*
EMO_g1959
EIN_241340
836
EMO_g5616
996
EHI_180650
993
EDI_057770
921
EMO_g986
EHI_097900
995
EIN_152250
EDI_099290
989
999
EHI_181230
408
EMO_g6079
759
EIN_277820
EIN_315880
834
EIN_140010
516
EIN_245400
589
EIN_210450
489
EIN_162840
EIN_162850
586
512
EIN_103270
EIN_114990
964
EIN_199850
EIN_050320

f)

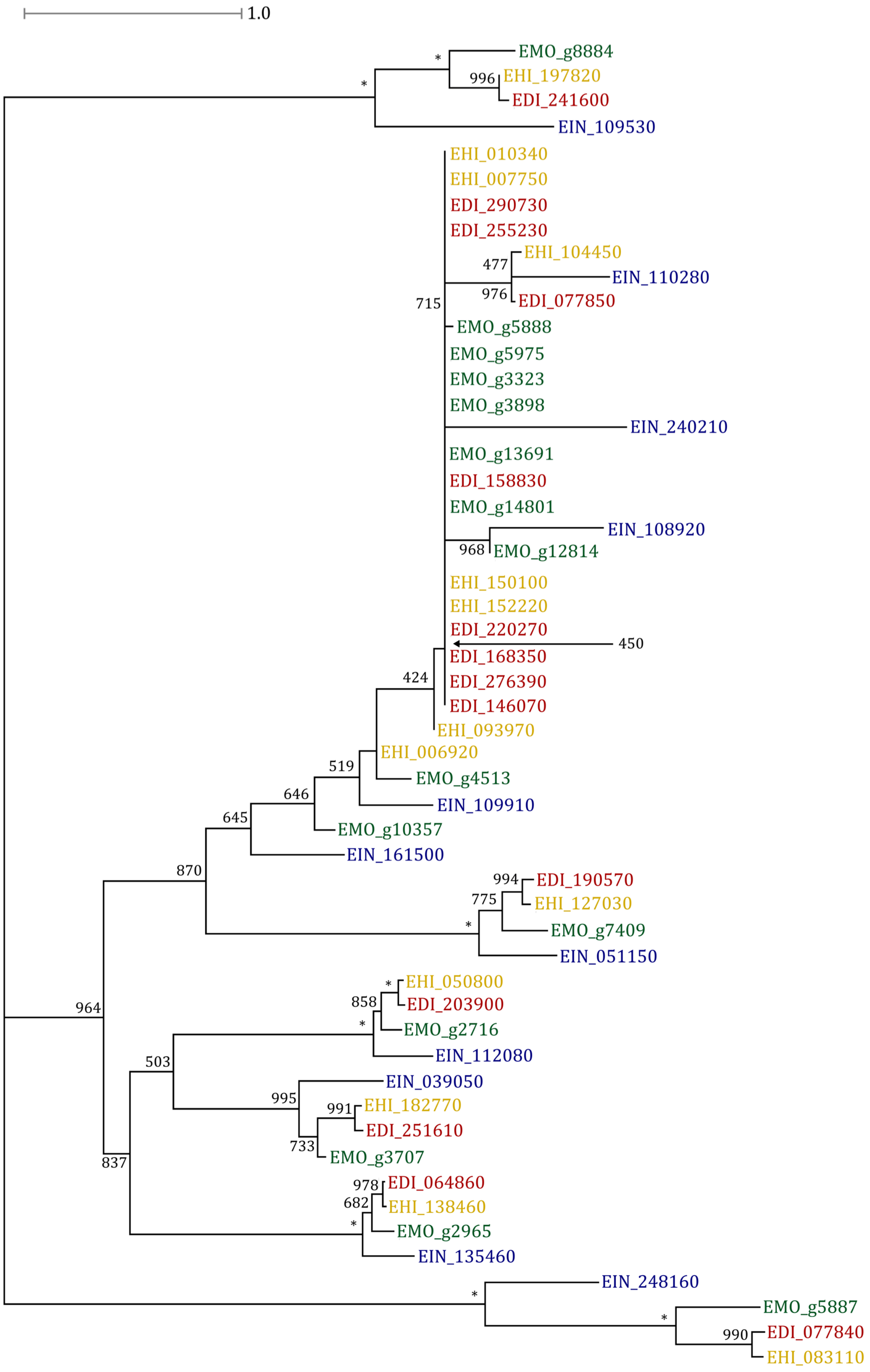
1.0
EMO_g8884
EHI_197820
EDI_241600
EIN_109530
EHI_010340
EHI_007750
EDI_290730
EDI_255230
EHI_104450
EIN_110280
EDI_077850
EMO_g5888
EMO_g5975
EMO_g3323
EMO_g3898
EIN_240210
EMO_g13691
EDI_158830
EMO_g14801
EIN_108920
EMO_g12814
EHI_150100
EHI_152220
EDI_220270
EDI_168350
EDI_276390
EDI_146070
EHI_093970
EHI_006920
EMO_g4513
EIN_109910
EMO_g10357
EIN_161500
EDI_190570
EHI_127030
EMO_g7409
EIN_051150
EHI_050800
EDI_203900
EMO_g2716
EIN_112080
EIN_039050
EHI_182770
EDI_251610
EMO_g3707
EDI_064860
EHI_138460
EMO_g2965
EIN_135460
EIN_248160
EMO_g5887
EDI_077840
EHI_083110
*
996
477
976
715
968
450
424
519
646
645
870
994
775
964
858
503
995
991
733
837
978
682
990

Fig S3. Cumulative divergence of *Entamoeba histolytica* and *Entamoeba moshkovskii* strains, relative to their reference strains, as a function of genotype quality up to values of '99'. *E. histolytica* strains are denoted by solid lines and *E. moshkovskii* strains by dotted lines.

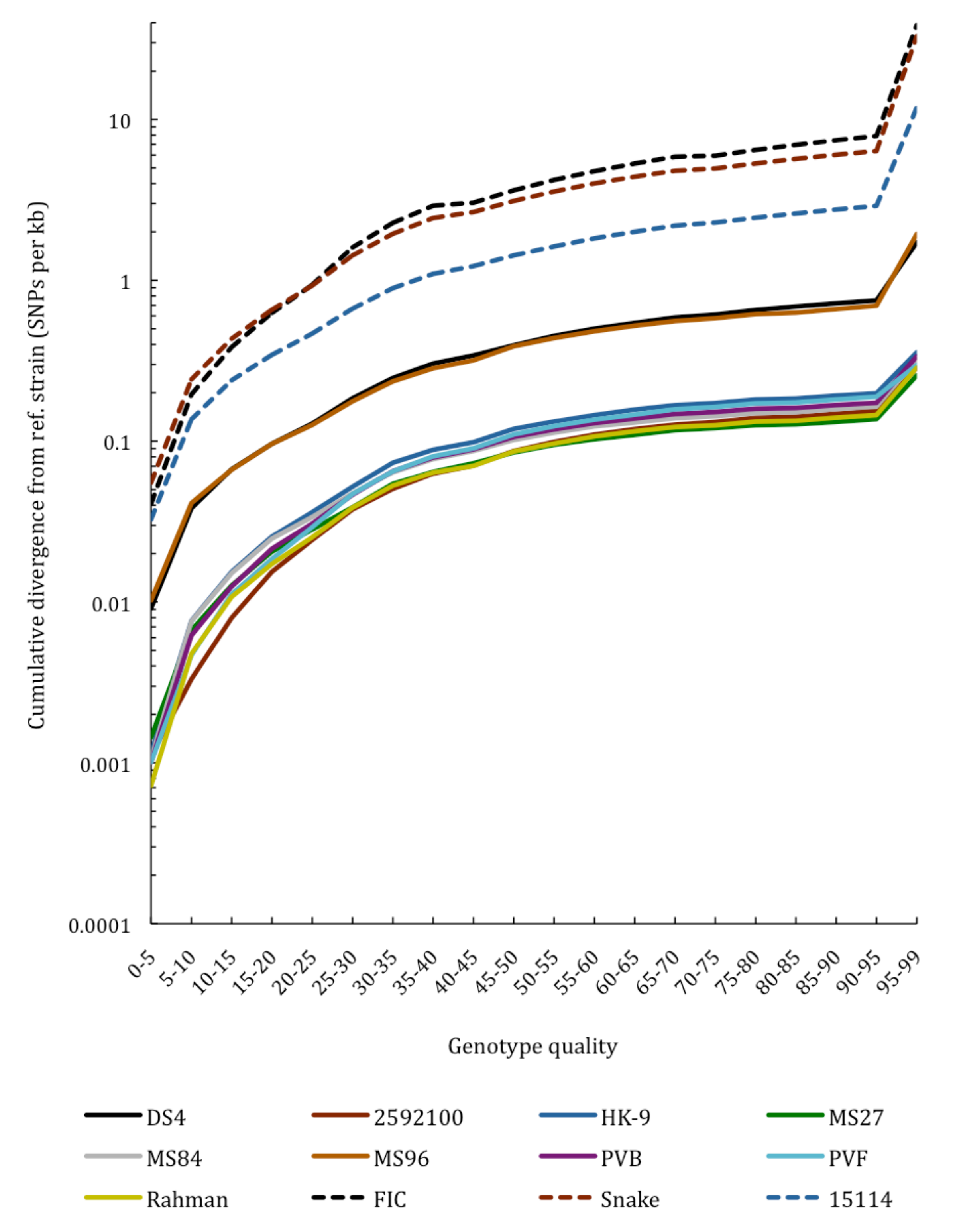

Fig S4. Probability-distributed log ratios of diversity in 2,485 *Entamoeba histolytica* and *Entamoeba moshkovskii* orthologue pairs. Ten *E. histolytica* and four *E. moshkovskii* strains were compared. Columns indicate observed counts of pairs at different diversity ratios. Red line represents normal distribution expected in the case of equal diversity of values within 3 standard deviations of the mean.

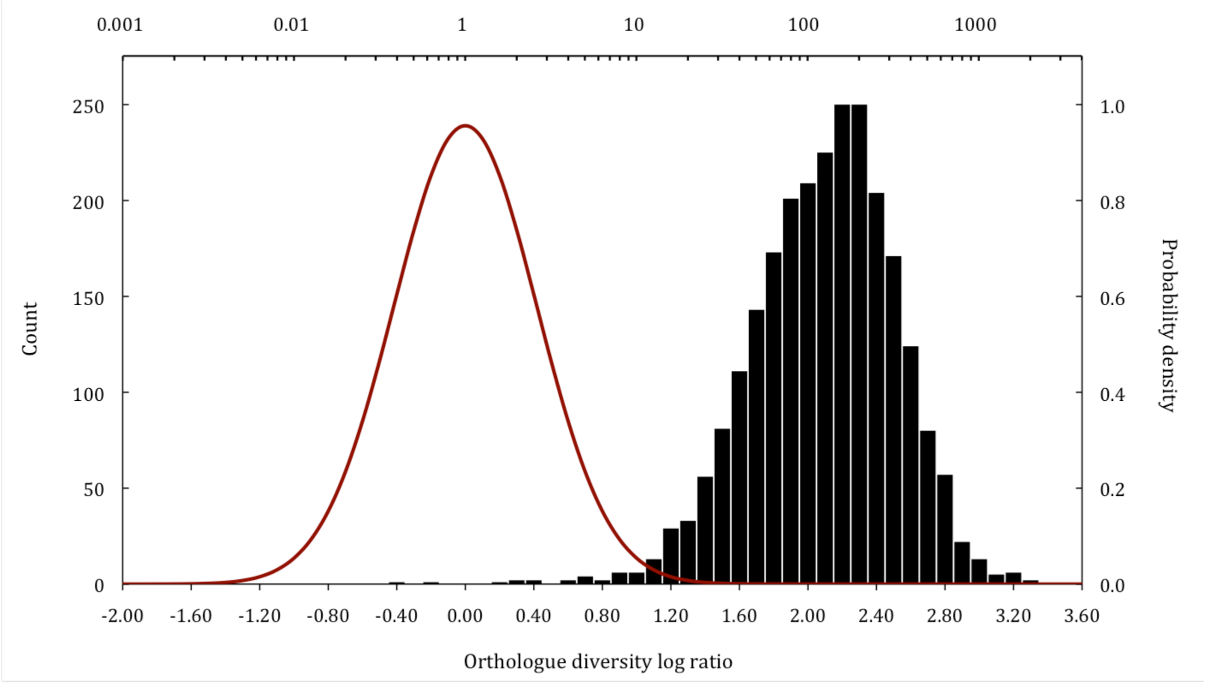

Fig S5. The proportion of 4-haplotype SNP pairs in *Entamoeba moshkovskii* as a function of the distance between the pairs. SNP pairs were physically linked (i.e. they were on the same scaffold/contig). Each point represents 1,000 randomly selected SNP pairs. The line represents the correlation between distance between pairs and proportions of 4-haplotype SNP pairs. The correlation was statistically insignificant.

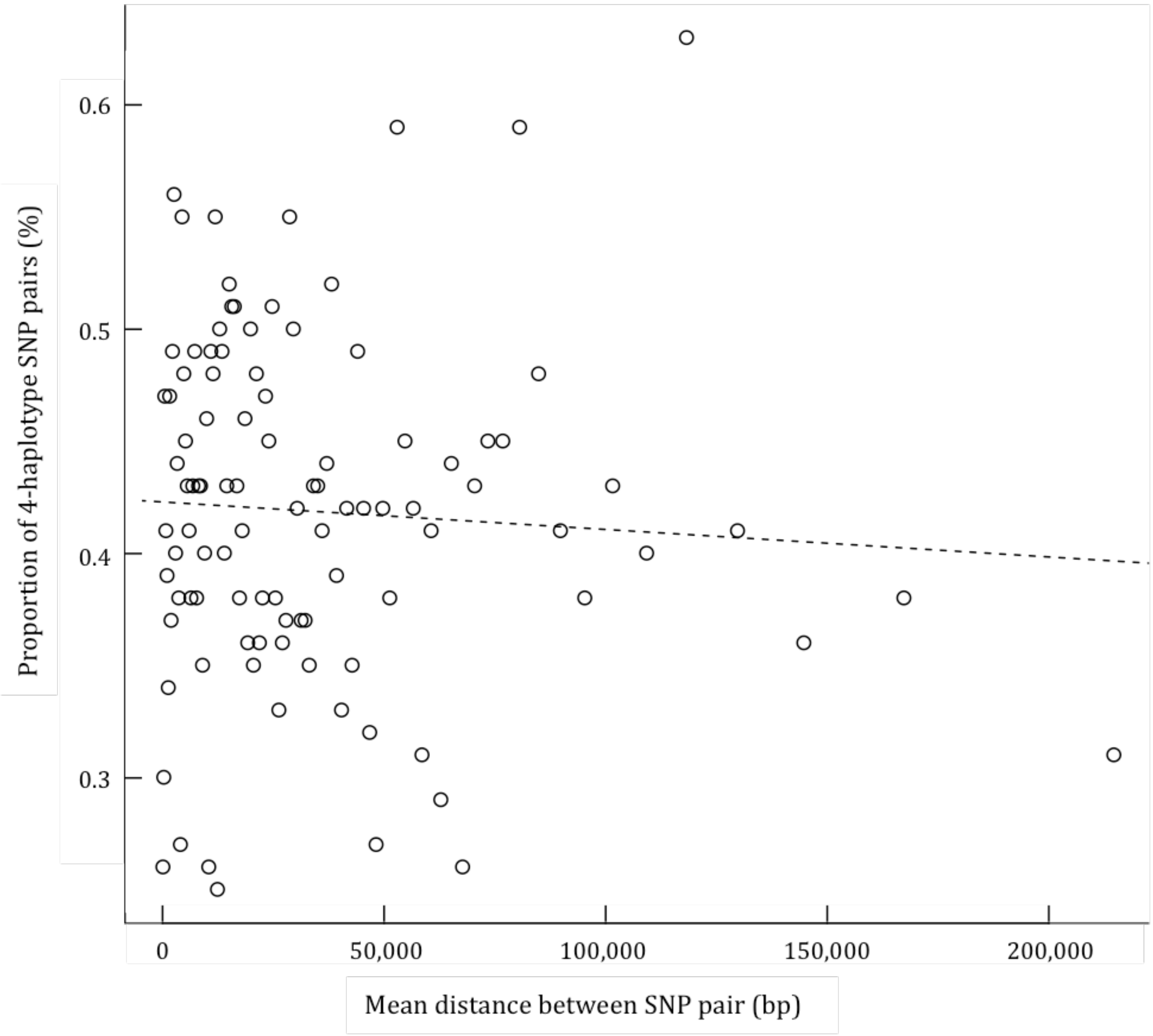

Table S1. Counts and proportions of variants detected when reads from the reference strains were mapped to existing reference genomes

Species	Homozygous SNPs		Heterozygous SNPs		Indels
	Count	SNPs/Kb	Count	SNPs/Kb	Count
E. histolytica	120	5.77 e-3	1,361	6.54 e-2	176
E. moshkovskii	165	6.53 e-3	33,527	1.33	2,446

Table S2. Functions under diversifying selective pressure in *E. moshkovskii* strains, according to dN/dS ratios, relative to the reference strain. Numbers represent number of genes to which each annotation was attributed within each strain.

Function	Strain		
	FIC	Snake	15114
BspA family	20	33	27
Serine/threonine/tyrosine? protein kinase	5	5	7
Surface antigen ariel1	-	-	2
Serine protease inhibitor/leukocyte elastase inhibitor	1	1	2
DNA double-strand break repair Rad50 ATPase	6	7	8
Heat shock protein 70	-	-	2
Cullin family protein	-	1	-
Ras family GTPase	1	2	2
PQ loop repeat protein	-	1	-
DEAD/DEAH box helicase	-	2	-
Nucleoside diphosphate kinase	1	-	1
Caldesmon	-	-	1
CAAX amino terminal protease family protein	-	-	1
Methyltransferase trm13 protein*	-	-	1
AAA family ATPase	-	-	1
Actin	-	2	2
Transitional endoplasmic reticulum ATPase	-	1	-
Mucin-2	-	-	1
Myosin heavy chain	-	-	1
Proline synthetase associated protein	-	-	1
Acetyltransferase, GNAT family	-	-	1
Hypothetical protein	19	35	70

Table S3. Functions under diversifying selection in *E. histolytica* strains, according to dN/dS ratios, relative to the reference strain. Numbers represent number of genes to which each annotation was attributed within each strain.

Function	Strain							
	**DS4**	**2592100**	**MS27**	**MS84**	**MS96**	**PVB**	**PVF**	**Rahman**
AIG1	-	-	-	1	1	-	-	-
Ser/Thr Protein Kinase	1	-	-	-	1	-	-	-
BspA	-	-	-	-	3	-	-	-
DNA polymerase	-	-	-	-	1	-	-	-
Regulator of nonsense transcripts	-	-	-	-	1	-	-	-
DEAD/DEAH box helicase	-	-	-	-	1	-	-	-
Hypothetical protein	3	1	1	1	6	2	2	1
