# Supplementary material for: Genetic Diversity and Gene Family Expansions in Members of the Genus Entamoeba
Source: Genome Biol Evol. 2019 Jan 21;11(3):688–705. doi: 10.1093/gbe/evz009 (PMC6414313; doi:10.1093/gbe/evz009)
Supplement: Supplementary Data [file evz009_supp.zip › Supporting information legends.docx]

**Fig S1. Frequencies of mean read depths within each scaffold/contig in the *Entamoeba moshkovskii* Laredo genome.** The highest fold coverage recorded was 7730.04x.

**Fig S2. Phylograms of *Entamoeba* gene families directly involved in virulence that demonstrate differential expansions and reductions across species.** Red boxes highlight clades in which pseudogenes were identified. They are linked to red boxes showing the same clades when phylogeny was calculated using nucleotide sequences, including the pseudogenes. Scale bars in red boxes represent nucleotide phylograms. All phylograms are midpoint rooted. Bootstrapping was performed for 1,000 replicates. Bootstrap values of 1,000 are represented by asterisks (*). Bootstrap values below 400 are not shown. a) Heavy Gal/GalNAc lectin subunits; b) Intermediate Gal/GalNAc lectin subunits; c) Light Gal/GalNAc lectin subunits; d) Cysteine protease Family A; e) Cysteine protease Family B; f) Cysteine protease Family C.

**Fig S3. Cumulative divergence of *Entamoeba histolytica* and *Entamoeba moshkovskii* strains, relative to their reference strains, as a function of genotype quality up to values of ‘99’.** *E. histolytica* strains are denoted by solid lines and *E. moshkovskii* strains by dotted lines.

**Fig S4. Probability-distributed log ratios of diversity in 2,485 *Entamoeba histolytica* and *Entamoeba moshkovskii* orthologue pairs.** Ten *E. histolytica* and four *E. moshkovskii* strains were compared. Columns indicate observed counts of pairs at different diversity ratios. Red line represents normal distribution expected in the case of equal diversity of values within 3 standard deviations of the mean.

**Fig S5 The proportion of 4-haplotype SNP pairs in *Entamoeba moshkovskii* as a function of the distance between the pairs.** SNP pairs were physically linked (i.e. they were on the same scaffold/contig). Each point represents 1,000 randomly selected SNP pairs. The line represents the correlation between distance between pairs and proportions of 4-haplotype SNP pairs. The correlation was statistically insignificant.
